# Chronic intake of high dietary sucrose induces sexually dimorphic metabolic adaptations in mouse liver and adipose tissue

**DOI:** 10.1038/s41467-022-33840-6

**Published:** 2022-10-13

**Authors:** Erin J. Stephenson, Amanda S. Stayton, Aarti Sethuraman, Prahlad K. Rao, Alice Meyer, Charles Klazer Gomes, Molly C. Mulcahy, Liam McAllan, Michelle A. Puchowicz, Joseph F. Pierre, Dave Bridges, Joan C. Han

**Affiliations:** 1 Department of Anatomy, College of Graduate Studies, Midwestern University, Downers Grove, IL 60515 USA; 2 Department of Pediatrics, College of Medicine, University of Tennessee Health Science Center, Memphis, TN 38103 USA; 3 Children’s Foundation Research Institute, Le Bonheur Children’s Hospital, Memphis, TN 38103 USA; 4 Department of Nutritional Sciences, University of Michigan School of Public Health, Ann Arbor, MI 48109-2029 USA; 5 Department of Microbiology and Immunology, College of Medicine, University of Tennessee Health Science Center, Memphis, TN 38164 USA; 6 Department of Nutritional Sciences, College of Agricultural and Life Sciences, University of Wisconsin-Madison, Madison, WI 53706 USA; 7 Department of Physiology, College of Medicine, University of Tennessee Health Science Center, Memphis, TN 38164 USA; 8 Department of Pediatrics, Icahn School of Medicine at Mount Sinai and Kravis Children’s Hospital, New York, NY 10029 USA

**Keywords:** Fat metabolism, Transcriptomics, Metabolic syndrome, Fatty acids, Non-alcoholic fatty liver disease

## Abstract

Almost all effective treatments for non-alcoholic fatty liver disease (NAFLD) involve reduction of adiposity, which suggests the metabolic axis between liver and adipose tissue is essential to NAFLD development. Since excessive dietary sugar intake may be an initiating factor for NAFLD, we have characterized the metabolic effects of liquid sucrose intake at concentrations relevant to typical human consumption in mice. We report that sucrose intake induces sexually dimorphic effects in liver, adipose tissue, and the microbiome; differences concordant with steatosis severity. We show that when steatosis is decoupled from impairments in insulin responsiveness, sex is a moderating factor that influences sucrose-driven lipid storage and the contribution of de novo fatty acid synthesis to the overall hepatic triglyceride pool. Our findings provide physiologic insight into how sex influences the regulation of adipose-liver crosstalk and highlight the importance of extrahepatic metabolism in the pathogenesis of diet-induced steatosis and NAFLD.

## Abstract (web summary)

Dietary sugar intake may contribute to the development non-alcoholic fatty liver disease. Here the authors investigated the effects of chronic dietary sucrose on the liver-adipose-microbiome axis in mice, and report that sex is a moderating factor that influences sucrose-driven lipid storage in the liver and adipose tissue lipolysis.

## Introduction

Non-alcoholic fatty liver disease (NAFLD) is the most common form of chronic liver disease, affecting ~25% of Earth’s human population^1^. NAFLD is characterized by the presence of >5% steatosis (simple steatosis), with or without inflammation and/or scarring in the liver (nonalcoholic steatohepatitis; NASH)^2^. Simple steatosis progresses to NASH in 59% of patients^1^, whereas advancing fibrosis in NASH can lead to cirrhosis, hepatocellular carcinoma, and liver failure^2^. Approximately 50% of all NAFLD patients have obesity^1^, and individuals with NAFLD have a higher incidence of type 2 diabetes, cardiovascular disease, and all-cause mortality^1,3,4^. However, not all patients with NAFLD have other metabolic comorbidities (and vice-versa), suggesting that although NAFLD and other metabolic disease states are related, their etiology can be decoupled^5^.

Findings from observational studies and meta analyses indicate that excessive sugar intake or compromised sugar metabolism may be an initiating factor for NAFLD in humans^6–11^. Although dietary sugars have been used extensively to induce cardiometabolic abnormalities in animal models^7,12–19^, the molecular mechanisms underlying sugar-induced hepatic lipid accumulation remain poorly understood in a context that is translational to human dietary practices^20^. Most preclinical studies rely upon use of supraphysiologic concentrations of sucrose, glucose, or fructose to induce steatosis and other metabolic comorbidities^19–22^, often with the confounding addition of excess dietary lipid^16,19,21^. This lack of translatability, in combination with reports that the effects of dietary sugars are dependent on physical form (i.e., liquid versus solid)^17,18^, make it difficult to draw mechanistic conclusions applicable to how sugar intake leads to NAFLD in humans.

Nonetheless, it has been generally accepted that high sucrose consumption (or consumption of the not-meaningfully different high-fructose corn syrup) causes an elevation in de novo fatty acid synthesis (and, subsequently, hepatic triglyceride storage) due to the ability of fructose, one half of the sucrose disaccharide, to proceed directly toward de novo fatty acid synthesis^23,24^. However, tracer studies at physiologic fructose concentrations indicate that only a small percentage (<1%) of ingested fructose is directly converted to lipid (most becomes glucose or lactate)^25,26^. Thus, in addition to fructose-driven fatty acid synthesis in liver, the development of steatosis following high sucrose intake likely involves fatty acids derived from extrahepatic sources^27–29^.

Most circulating non-esterified fatty acids (NEFA) are derived from adipose tissue lipolysis^30^. Adipose tissue mass increases rapidly in response to sucrose intake^13,16,21^, whereas increased adiposity following sucrose feeding in humans^31^ and rats^22^ leads to an increase in the rate of adipose tissue lipolysis ex vivo. Thus, in addition to direct effects on liver, high dietary sucrose intake induces molecular changes in adipose tissue that lead to dysregulated lipolysis and a subsequent increase circulating NEFA which would, in turn, be expected to increase demand for fatty acid uptake and re-esterification in the liver.

Here, we investigated the effects of chronic, physiologically relevant dietary sucrose intake on energy balance and the liver-adipose axis. We hypothesized that chronic sucrose intake would drive hepatic steatosis indirectly, primarily as a result of dysregulated adipose tissue lipolysis and increased re-esterification of extrahepatic-derived fatty acids in the liver. Additionally, because there are a lack of in vivo data to support epidemiological data^32,33^ and in silico modeling^34,35^ suggesting that NAFLD develops through distinct metabolic processes in males and females, we also sought to determine how sex impacts the severity of sucrose-induced steatosis and other metabolic parameters that may be affected by chronic liquid sucrose intake.

## Results

### Sucrose increases adiposity

We *ad libitum* fed male and female mice standard rodent chow (3.1 kCal/g, 17% kCal from fat, 58% from carbohydrate, 25% from protein) with water from weaning until 10 weeks of age, after which mice continued to receive the chow diet with water (control groups) or chow with water containing 10% w/v sucrose (treatment groups) for 12 weeks.

**Fig. 1:**
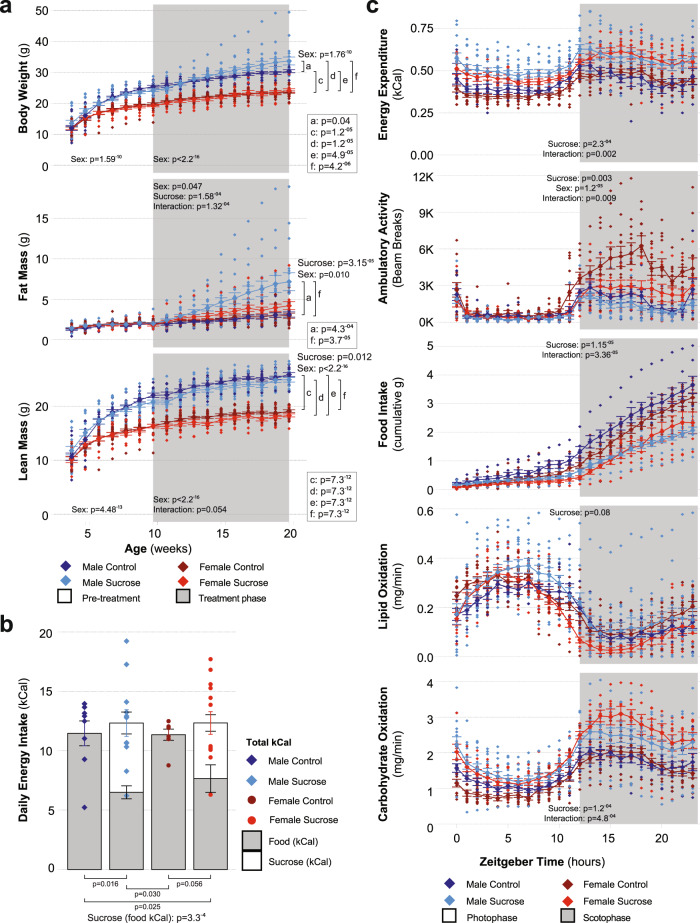
Sucrose intake modulates macronutrient metabolism and storage. **a** Longitudinal effects of sucrose intake on total body mass, fat mass, and lean mass as determined by linear mixed effects models with likelihood ratio tests. 20-week (10 weeks treatment) data were log-transformed and compared by two-way ANOVA with post hoc Tukey’s test (fat and lean mass) or Scheirer–Ray–Hare test and post-hoc Wilcoxon Rank Sum test with continuity correction (body weight; MC: *n* = 15, MS: *n* = 18, FC: *n* = 19, FS: *n* = 13; see figure for *p*-values). **b** Caloric intake from food was reduced in the sucrose groups as determined by Scheirer–Ray–Hare test and post hoc Dunn analysis with Benjamini and Hochberg correction for multiple comparisons (MC: *n* = 8, MS: *n* = 11, FC: *n* = 7, FS: *n* = 13; see figure for *p*-values). **c** 24-h energy expenditure, ambulatory activity, cumulative food intake, lipid oxidation, and carbohydrate oxidation were all also modified by sucrose intake as determined by linear mixed effects models with likelihood ratio tests (MC: *n* = 7, MS: *n* = 14, FC: *n* = 12, FS: *n* = 9; see figure for *p*-values). Data displayed are the group means ± standard error with biologically independent values overlayed. MC = Male Control, MS = Male Sucrose, FC = Female Control, FS = Female Sucrose. Source data are provided in the source data file.

Prior to the treatment period (4–10 weeks of age, Fig. 1a, unshaded area), we observed a main effect of sex on body weight (*p* = 1.59^−10^, upper panel) and lean mass gains (*p* = 4.48^−13^, lower panel), but not fat mass gains (*p* = 0.582, center panel). Treatment designation had no effect on any of the body composition parameters (*p* = 0.441 for body weight, *p* = 0.885 for body fat and *p* = 0.371 for lean mass).

During the treatment period (10 weeks of age onward; Fig. 1a, shaded area), sucrose intake resulted in increased body fat (*p* = 1.58^−4^), whereas no effect of sucrose was observed for total body weight (*p* = 0.114) or lean mass (*p* = 0.897). An effect of sex was observed for weight (*p* < 2.20^−16^), fat (*p* = 0.047), and lean mass (*p* < 2.20^−16^). We also observed a sex:sucrose interaction for body fat (*p* = 1.32^−4^), but not weight (*p* = 0.233) or lean mass (*p* = 0.054). By 20 weeks of age, sucrose intake had an effect on body fat (*p* = 3.15^−5^), with increased body weight in male (10.9%, *p* = 0.037) but not female mice (*p* = 0.544) observed. Increased weight in males following sucrose intake was associated with an increase in body fat (129.7%, *p* = 4.31^−4^). We continued to observe an effect of sex on body weight (*p* = 1.76^−10^), on body fat (*p* = 0.010), and lean mass (*p* < 2.2^−16^) during this time.

To determine the effect of sucrose intake on specific adipose tissue depots, we carefully dissected out and weighed both subcutaneous (iWAT) and visceral (gWAT) depots (Supplementary Fig. 1a). No effect of sex was observed for either inguinal (*p* = 0.731) or gonadal (*p* = 0.084) adipose tissue, although we did detect an effect of sucrose intake (inguinal: *p* = 6.6^−4^, gonadal: *p* = 5.5^−4^). In both males and females, sucrose intake increased the mass of inguinal fat (88.2%, *p* = 0.031, and 55.6%, *p* = 0.049, respectively). Sucrose intake also increased gonadal fat mass in males (84.0%, *p* = 0.009), although in females the increase in gonadal fat mass did not attain statistical significance (49.1%, *p* = 0.085). Together, these results demonstrate that chronic sucrose intake increases adiposity and that the effect of sucrose on adiposity gains is similar in males and females.

### Sucrose intake modifies energy balance

To determine the role of energy intake in determining adiposity gains following chronic sucrose intake, we measured the average daily food and fluid intake of mice individually housed between weeks 10–11 of treatment (Fig. 1b). Sucrose intake led to reduced energy intake from food (Fig. 1b, shaded bars; *p* = 3.3^−4^) with a reduction observed in males (−43.3%; *p* = 0.016). Reduced food intake in females was also observed; however, this did not attain statistical significance (−32.6%; *p* = 0.056). Total energy intake was similar between all four groups regardless of sex or treatment designation (Fig. 1b). Notably, absolute fluid intake was elevated in response to sucrose intake (*p* = 8.5^−5^) but not sex (*p* = 0.273), with increased intake of fluid in both male (194.9%, *p* = 0.005) and female mice (182.9%, *p* = 0.039) if sucrose was present in the drinking water.

To determine the relative contribution of energy expenditure to adiposity gains following liquid sucrose intake, we performed indirect calorimetry and activity monitoring experiments in parallel with measurements of energy intake beginning at 20 weeks of age (10 weeks of sucrose treatment). Sex had no effect on energy expenditure (*p* = 0.343), whereas sucrose intake increased energy expenditure in both sexes (Fig. 1c, upper panel; *p* = 2.3^−4^). A sex:sucrose interaction was also observed (*p* = 0.002), with males appearing to have a greater increase in energy expenditure than females after sucrose intake. Changes in physical activity did not explain the increase in energy expenditure observed following sucrose intake, as ambulatory activity was decreased in both sexes in response to sucrose intake (*p* = 0.003), primarily during the scotophase (shaded area of Fig. 1c, second panel), with a sex:sucrose interaction also observed (*p* = 0.009). To establish whether changes in energy substrate oxidation might be contributing to sucrose-induced increases in adiposity, we calculated the rates of both lipid (fourth panel of Fig. 1c) and carbohydrate oxidation (last panel of Fig. 1c), observing effects of sucrose intake on carbohydrate oxidation (*p* = 1.2^−4^), but not lipid oxidation (*p* = 0.08), with females appearing to have greater magnitude increases in carbohydrate oxidation than males following sucrose intake. To establish the effects of sucrose intake on energy balance in the absence of thermal stress, we repeated these experiments with animals housed at the lower end of their thermoneutral zone (28 °C; Supplementary Fig. 1b). An effect of sex was only observed for ambulatory activity (*p* = 4.0^−4^), whereas sucrose intake continued to suppress both food intake (2.63^−7^) and ambulatory activity (*p* = 0.037), with a sucrose:sex interaction observed for food intake (*p* = 3.12^−7^). Under thermoneutral conditions, neither sex nor sucrose intake influenced energy expenditure (*p* = 0.172 and *p* = 0.858, respectively), lipid oxidation (*p* = 0.833 and *p* = 0.536, respectively), or carbohydrate oxidation (*p* = 0.429 and *p* = 0.438, respectively).

To determine whether impairments in glucose uptake or insulin responsiveness were contributing to sucrose-induced changes in substrate utilization we performed insulin and glucose tolerance tests (Supplementary Fig. 2a–d). While an effect of sex was observed for both tests (*p* = 0.003 for insulin tolerance test, *p* = 7.4^−4^ for glucose tolerance test), sucrose intake had no effect on either parameter. Neither sex nor sucrose intake affected fasting blood glucose concentrations (Supplementary Fig. 2e), whereas there was an effect of sex on fasting serum insulin (Supplementary Fig. 2f; *p* = 1.1^−4^), with 26.5% lower concentrations observed in the female control group compared to the male control group (*p* = 7.4^−4^). Serum corticosterone was similar across all four groups (Supplementary Fig. 2g). Taken together, our findings for energy balance and glucose homeostasis suggest there may be a disconnect between energy intake, energy expenditure, and adiposity gains that occur in the absence of systemic insulin resistance following chronic liquid sucrose intake.

### Sucrose induces hepatic steatosis via sex-specific mechanisms

**Fig. 2:**
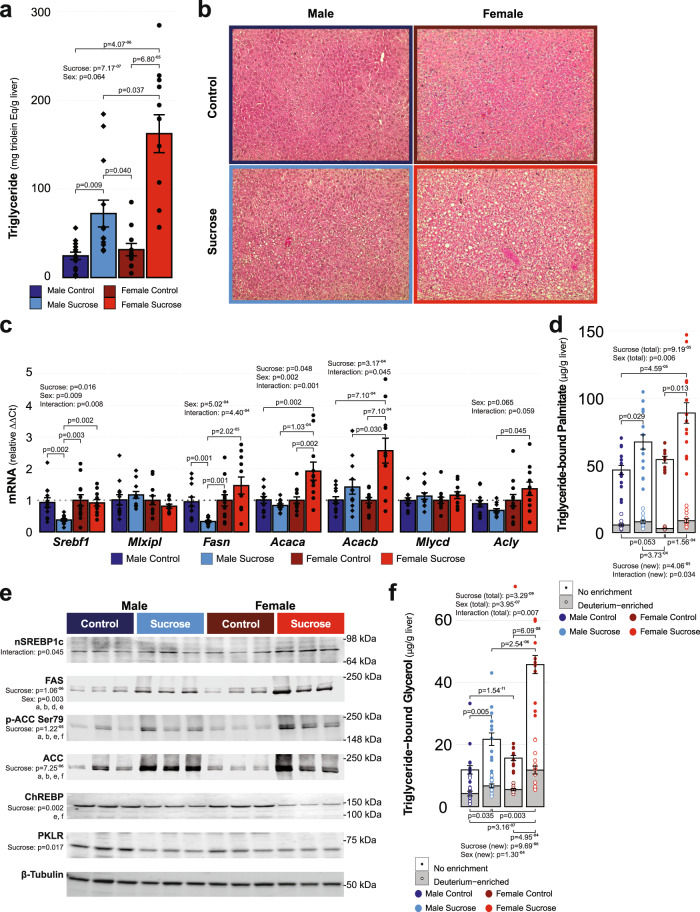
Sucrose intake increases hepatic triglyceride content via sex-specific mechanisms. **a** Sucrose intake increased hepatic triglyceride content in both sexes; however, the magnitude of the sucrose-driven increase was greatest in female mice, as determined by Scheirer–Ray–Hare test and post hoc Dunn analysis with Benjamini and Hochberg correction for multiple comparisons (MC: *n* = 14, MS: *n* = 13, FC: *n* = 11, FS: *n* = 12; see figure for *p*-values). **b** Representative hematoxylin and eosin staining of livers. **c** Transcriptional regulation of fatty acid synthesis also exhibited sex-specific effects of sucrose intake as determined in log-transformed data using two-way ANOVA with post hoc Tukey’s tests (MC: *n* = 12, MS: *n* = 12, FC: *n* = 12, FS: *n* = 11; see figure for *p*-values). **d** Transcriptional data were supported by mass spectrometry data demonstrating that sucrose intake increased the total pool of triglyceride-bound palmitate in both sexes, but only increased newly synthesized palmitate in females, as determined in log-transformed data using two-way ANOVA with post hoc Tukey’s tests (MC: *n* = 14, MS: *n* = 14, FC: *n* = 10, FS: *n* = 13; see figure for *p*-values). **e** Representative western blots demonstrating that the abundance of proteins encoded by the transcripts differentially expressed (as well as ChREBP and its target PKLR) between males and female mice in response to sucrose intake. Statistics were derived following densitometric quantification of bands from samples run on a single gel that were normalized to total protein and then log transformed and compared using two-way ANOVA with post hoc Tukey’s tests (FAS: ^a^*p*= 1.43^−4^, ^b^*p* = 1.55^−4^, ^d^*p* = 0.044, ^e^*p* = 2.80^−6^; pACC: ^a^*p* = 0.001, ^b^*p* = 0.007, ^e^*p* = 2.8^−4^, ^f^*p* = 0.041; ACC: ^a^*p* = 0.001, ^b^*p* = 0.003, ^e^*p* = 2.99^−4^, ^f^*p* = 0.014; ChREBP: ^e^*p* = 0.024, ^f^*p* = 0.010; MC: *n* = 6, MS: *n* = 6, FC: *n* = 6, FS: *n* = 6). **f** Total glycerol and glycerol from glyceroneogenesis were increased in both sexes in response to sucrose intake, with the magnitude of these increases greater in female mice as determined in transformed data using two-way ANOVA with posthoc Tukey tests (MC: *n* = 14, MS: *n* = 14, FC: *n* = 10, FS: *n* = 13). Data displayed are the group means ± standard error with biologically independent values overlayed. MC Male Control, MS Male Sucrose, FC Female Control, FS Female Sucrose. Source data, including uncropped and unprocessed blots, are provided in the source data file.

We first confirmed that chronic liquid sucrose intake at physiologically relevant concentrations resulted in hepatic steatosis by quantifying liver triglyceride content biochemically (Fig. 2a) and visualizing liver histology (Fig. 2b; representative image after staining with hematoxylin and eosin). An effect of sucrose intake (*p* = 7.17^−7^), but not quite sex (*p* = 0.064) was observed for total triglyceride content. Sucrose intake increased liver triglycerides in both males (194.4%, *p* = 0.009) and females (415.2%, *p* = 6.8^−5^) compared to their corresponding control groups, with female mice in the sucrose group having an exacerbated response compared to males (124.8%, *p* = 0.037).

To identify what might be causing these differences at the transcriptional level, we measured the expression of a selection of transcripts known to regulate de novo fatty acid synthesis (Fig. 2c; Supplementary Data File 1). An effect of sex was observed for several transcripts, including: *Srebf1* (*p* = 0.009), *Fasn* (*p* = 5.02^−4^), and *Acaca* (*p* = 0.002). An effect of sucrose intake was observed for *Srebf1* (*p* = 0.016), *Acaca* (*p* = 0.048) and *Acacb* (*p* = 3.17^−4^), while the effect of sucrose intake did not attain statistical significance for *Fasn* (*p* = 0.066). In response to sucrose intake, males had reduced expression of *Srebf1* and *Fasn* (59.4%, *p* = 0.002 and 65.4%, *p* = 0.001; respectively), whereas females had increased expression of *Acaca* and *Acacb* (92.2%, *p* = 0.002 and 157.1%, *p* = 2.02^−5^; respectively). Sex:sucrose interactions were observed for *Srebf1* (*p* = 0.008), *Fasn* (*p* = 4.4^−4^), *Acaca* (*p* = 0.001) and *Acacb* (*p* = 0.045). *Acly* also showed a tendency toward a sex:sucrose interaction, although this did not attain statistical significance (*p* = 0.059).

Since the transcript data suggested that differences in de novo fatty acid synthesis may be driving some of the sex differences we observed in response to sucrose intake, we used a deuterium enrichment strategy to measure the amount of newly synthesized palmitate in the hepatic triglyceride pool (Fig. 2d). We observed both sex (*p* = 0.006) and sucrose intake effects (*p* = 9.19^−5^) for total triglyceride-bound palmitate (Fig. 2d, open bars/solid points). Both males and females had increased total triglyceride-bound palmitate following sucrose intake compared to their corresponding control groups (44.7%, *p* = 0.029 and 64.9%, *p* = 0.013; respectively). Of the total pool of triglyceride-bound palmitate, the contribution made by newly synthesized palmitate (Fig. 2d, gray inlaid bars/open points) showed an effect of sucrose intake (*p* = 4.06^−5^), and a sucrose:sex interaction effect (*p* = 0.034), with newly synthesized palmitate only increased in female mice after sucrose intake (66.4%, *p* = 1.56^−4^).

Given that de novo fatty acid synthesis can be regulated post-transcriptionally, we also measured the relative abundance of proteins encoded by the transcripts we identified as being differentially expressed between male and female mice following sucrose intake, and ChREBP, which although was not different at the transcriptional level (*Mlxipl*, see above), is known to be regulated at the protein level (Fig. 2e). FAS and the smaller of the two bands corresponding to ChREBP were the only proteins measured that demonstrated an effect of sex (*p* = 0.003 and *p* = 0.036), although the ratio of phosphorylated ACC1/2 (inhibitory phosphorylation) at Ser79 to total ACC also demonstrated an effect of sex (*p* = 0.033), while there was a sex:sucrose interaction for the abundance of nuclear SREBP1-c (*p* = 0.045). An effect of sucrose intake was observed for FAS (*p* = 1.06^−6^), the larger of the two bands corresponding to ChREBP (*p* = 0.002), phospho-ACC1/2 at Ser79 (*p* = 1.22^−5^) and total ACC1/2 (*p* = 7.25^−6^), with FAS, phospho-ACC1/2 at Ser79 and total ACC1/2 being increased in abundance in response to sucrose intake in both male (98.0%, ^a^*p* = 1.43^−4^; 223.0%, ^a^*p* = 0.001 and 178.4%, ^a^*p* = 0.001; respectively) and female mice (129.8%, ^b^*p* = 1.55^−4^; 225.3%, ^b^*p* = 0.007 and 223.1%, ^b^*p* = 0.003; respectively), while ChREBP tended to be decreased in both sexes (male −38.1%, female −30.0%) although these differences did not attain statistical significance (male, *p* = 0.107 and female, *p* = 0.089). Sucrose-induced increases in hepatic FAS protein were also greater in female compared to male mice (57.2%, ^d^*p* = 0.044), whereas although SREBP1-c appeared to be increased in females compared to males after sucrose intake, this was not significant (35.5%, *p* = 0.061). To be sure that ChREBP activity wasn’t influencing fatty acid synthesis and that SREBP1-c was the main driver of fatty acid synthesis in our model, we measured the expression of PKLR. PKLR is a direct target of ChREBP but is not involved in lipid synthesis. In line with our findings for ChREBP, an effect of sucrose intake on PKLR was observed (*p* = 0.017), with decreased expression observed only in male mice following sucrose intake (−28.6%) although this was not statistically significant (*p* = 0.220). Taken together, these data provide mechanistic insight into how chronic sucrose intake results in hepatic steatosis and identifies sex as a moderating factor that can determine the contribution of de novo fatty acid synthesis to the hepatic triglyceride pool. Specifically, we show that female mice have increased rates of de novo fatty acid synthesis which is regulated, at least in part, by Srebf1/SERBP1-c and the transcription factor’s targets, Fasn/FAS and Acaca/Acacb.

Since upregulation of de novo fatty acid synthesis was not the main factor driving the increase in liver triglyceride we observed following chronic liquid sucrose intake, we also determined the relative contribution made by fatty acid re-esterification by measuring glyceroneogenesis via deuterium incorporation into triglyceride-bound glycerol (Fig. 2f). We observed effects of sex (*p* = 3.95^−7^), sucrose intake (*p* = 3.29^−09^) and a sex:sucrose interaction (*p* = 0.007) for the total pool of triglyceride-bound glycerol (Fig. 2f, open bars/solid points). Both male (83.0%, *p* = 0.005) and female (192.1%, *p* = 6.09^−8^) mice receiving sucrose had an increase in the total pool of triglyceride-bound glycerol compared to their respective control groups, whereas female mice receiving sucrose had more glycerol than male mice receiving sucrose (110.7%, *p* = 2.54^−6^). Of the total pool of triglyceride-bound glycerol, the contribution made by newly synthesized glycerol (Fig. 2f, gray inlaid bars/open points) was dependent on both sex (*p* = 1.30^−4^) and sucrose intake (*p* = 9.69^−6^). Similar to our observations for the total glycerol pool, newly synthesized glycerol increased in response to sucrose intake in both males (58.7%, *p* = 0.035) and females (114.0%, *p* = 4.95^−4^), and this effect was greater in female mice receiving sucrose compared to males (75.6%, *p* = 0.003). Together, these findings suggest that hepatic triglyceride accumulation following chronic liquid sucrose intake is primarily driven by the re-esterification of fatty acids derived from extrahepatic sources in male mice, and by both de novo fatty acid synthesis and re-esterification of extrahepatic fatty acids in female mice.

After identifying an effect of sucrose intake for the hepatic expression of the transcript *Scd1* (*p* = 1.25^−08^), and an effect of sex (*p* = 0.031) and a sex:sucrose interaction effect for *Elovl6* (*p* = 0.026; Supplementary Fig. 3a), we leveraged the presence of deuterium labeling to determine the effect of sex and sucrose intake on fatty acid modifications and their contribution to the hepatic triglyceride pool, including the elongation of palmitate into stearate (Supplementary Fig. 3b), and desaturation of stearate into oleate (Supplementary Fig. 3c). Total triglyceride-bound stearate was affected by sex (Supplementary Fig. 3b, open bars/solid points, *p* = 0.001), whereas newly elongated stearate was increased by sucrose intake only (Supplementary Fig. 3b, gray inlaid bars/open points, *p* = 2.53^−6^), with increases observed in both males (37.4%, *p* = 0.023) and females (164.4%, *p* = 8.59^−5^) in response to sucrose intake. Total triglyceride-bound oleate was affected by both sex (Supplementary Fig. 3c, open bars/solid points, *p* = 5.34^−8^) and sucrose intake (*p* = 5.83^−13^), with female mice having more total oleate than male mice in both the control (68.0%, *p* = 0.008) and sucrose groups (119.1%, *p* = 9.38^−5^), and sucrose intake increasing total oleate in both male (204.1%, *p* = 1.11^−7^) and female mice (296.5%, *p* = 2.78^−8^). We observed an effect of sucrose intake on newly desaturated triglyceride-bound oleate (Supplementary Fig. 3c, gray inlaid bars/open points, *p* = 1.46^−8^); relative to the control groups, sucrose intake increased newly desaturated oleate in both male (297.2%, *p* = 0.003) and female mice (1382.9%, *p* = 3.02^−06^). Together, these data suggest both sex and sucrose intake moderate the modification of fatty acids and their incorporation into the hepatic triglyceride pool.

### Sucrose potentiates β-adrenergic receptor-stimulated lipolysis

**Fig. 3:**
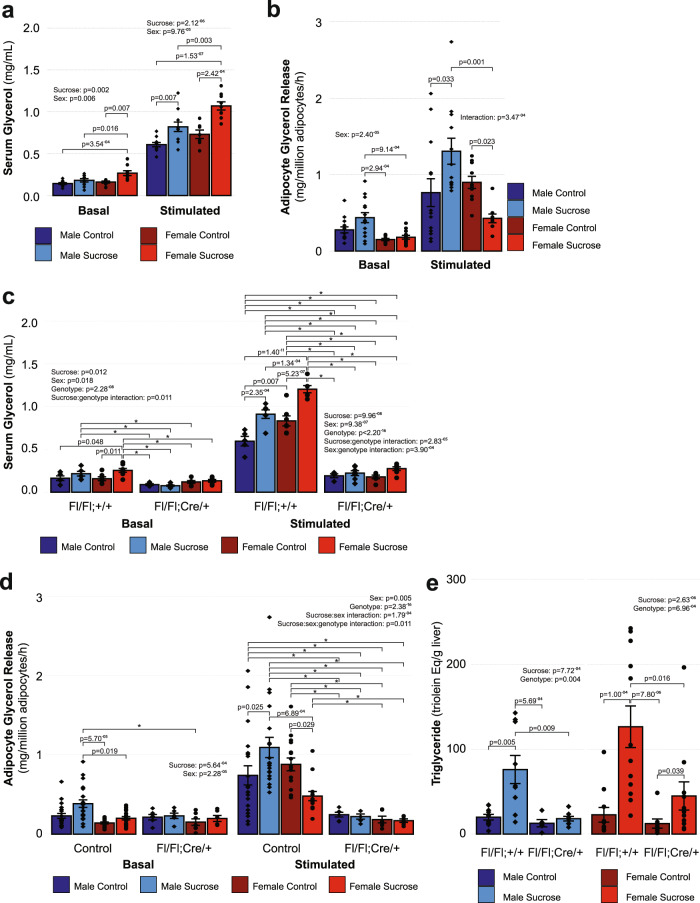
Limiting adipocyte lipolysis protects against sucrose-induced hepatic steatosis. **a** Serum glycerol appearance in response to in vivo β-AR stimulation with isoproterenol was increased in both sexes following chronic liquid sucrose intake as determined by two-way ANOVA with post-hoc Tukey’s test (MC: *n* = 11, MS: *n* = 11, FC: *n* = 7, FS: *n* = 9; see figure for *p*-values). **b** Sucrose intake potentiated stimulation-induced glycerol release from adipocytes from male mice but attenuated stimulated glycerol release in adipocytes from female mice as determined by two-way ANOVA with post hoc Tukey’s test (log-transformed basal values) and Scheirer–Ray–Hare test and post hoc Dunn analysis with Benjamini and Hochberg correction for multiple comparisons (stimulated values; basal/stimulated MC: *n* = 14/13, MS: *n* = 14/12, FC: *n* = 11/10, FS: *n* = 13/9; see figure for *p*-values). **c**, **d** Compared to mice with control genotypes (serum basal/stimulated MC: *n* = 5/5, MS: *n* = 6/6, FC: *n* = 7/7, FS: *n* = 8/8; adipocytes basal/stimulated MC: *n* = 21/20, MS: *n* = 21/19, FC: *n* = 17/16, FS: *n* = 19/14), β-AR stimulation did not potentiate release of glycerol in either serum (**c**) or isolated adipocytes (**d**) from mice with adiponectin-driven knock-out of adipose triglyceride lipase (serum basal/stimulated MC: *n* = 4/4, MS: *n* = 7/7, FC: *n* = 6/6, FS: *n* = 7/7; adipocytes basal/stimulated MC: *n* = 6/6, MS: *n* = 6/5, FC: *n* = 7/6, FS: *n* = 6/6) as determined by multifactorial ANOVA with post hoc Tukey’s test performed on absolute values (serum; *p < 0.05) or log transformed values (adipocytes; *p < 0.05). **e** The blunted adipocyte lipolysis response in knockout mice (**d**) prevented (males) or attenuated (females) sucrose-associated hepatic triglyceride accumulation as determined by multifactorial ANOVA with post hoc Tukey’s test (control/knockout MC: *n* = 10/5, MS: *n* = 9/8, FC: *n* = 11/8, FS: *n* = 12/11). Data displayed are the group means ± standard error with biologically independent values overlayed. MC Male Control, MS Male Sucrose, FC Female Control, FS Female Sucrose. Source data are provided in the source data file.

To determine if increased availability of fatty acids from extrahepatic sources was contributing to the increase in re-esterification and the subsequent steatosis we observed in response to chronic sucrose intake, we measured serum glycerol and NEFA in mice both before (basal) and 15 min after stimulating lipolysis in vivo with the non-specific β-adrenergic receptor (β-AR) agonist isoproterenol (stimulated; Fig. 3a and Supplementary Fig. 3d). For serum glycerol (representative of systemic lipolysis; Fig. 3a), we observed effects of both sex (*p* = 0.006) and sucrose intake (*p* = 0.002) under basal conditions, with female mice having 47.2% greater basal glycerol concentrations than males after sucrose intake (*p* = 0.016) and 66.2% greater glycerol concentrations compared to the female control group (*p* = 0.007). There were also effects of sex (*p* = 9.76^−5^) and treatment (*p* = 2.12^−6^) under β-AR-stimulated conditions, with sucrose intake potentiating the increase in serum glycerol concentrations in both males (35.1%, *p* = 0.0.007) and females (46.2%, *p* = 2.42^−4^), with greater glycerol concentrations being observed in females compared to males following sucrose intake (30.3%, *p* = 0.003). For serum NEFA (representative of both systemic lipolysis and fatty acid clearance/tissue uptake; Supplementary Fig. 3d), there were no effects of sex or treatment under basal conditions; however, under β-AR-stimulated conditions, there was a sex:treatment interaction effect (*p* = 0.029), with sucrose intake resulting in elevated serum NEFA concentrations in female mice when compared to males (12.7%, *p* = 0.031). No effect of sex (*p* = 0.051) or treatment (*p* = 0.394) was found.

### Sex moderates the adipocyte lipolysis response to β-AR stimulation

To test whether adipocyte-specific lipolysis was responsible for the potentiated induction of serum glycerol appearance we observed following systemic β-AR stimulation, we isolated mature adipocytes from iWAT and measured the rate of glycerol release under basal or β-AR-stimulated conditions (Fig. 3b). We observed an effect of sex (*p* = 2.40^−5^) under basal conditions, but no effect of sucrose intake (*p* = 0.077). Sucrose intake led to 141.9% greater basal adipocyte glycerol release in male mice than females (*p* = 9.14^−4^) and also tended to be higher in males in the control group compared to control females, however, the latter did not attain statistical significance (84.3%, *p* = 0.058). Under β-AR-stimulated conditions, a sex:sucrose interaction was observed (*p* = 3.47^−4^). Sucrose intake potentiated the effect of β-AR stimulation on lipolysis in adipocytes from male mice (70.9%, *p* = 0.033), whereas it attenuated the increase in lipolysis in adipocytes from female mice (−52.4%, *p* = 0.023). Stimulated lipolysis was also 205.6% greater in males than females after sucrose intake (*p* = 0.001). These findings suggest that in male mice, sucrose intake leads to increased adipocyte lipolysis via adipocyte-autonomous mechanisms whereas sucrose-induced potentiation of β-AR-dependent lipolysis in females may require a non-adipocyte intermediate to facilitate the systemic response.

### Inhibition of adipocyte lipolysis protects against hepatic steatosis

To confirm whether increased adipocyte lipolysis contributes to the development of hepatic steatosis following chronic liquid sucrose intake, we repeated our sucrose drinking paradigm in mice homozygous for a LoxP-modified *Pnpla2* allele^36^ with or without hemizygosity for an adipose tissue-specific Cre recombinase (*Adipoq*)^37^. *Pnpla2* encodes adipose triglyceride lipase (ATGL), the rate-limiting enzyme of lipolysis. Mice missing ATGL from their adipose tissue had a marked impairment in systemic β-AR-stimulated serum glycerol and NEFA appearance (Fig. 3c and Supplementary Fig. 3e), and the β-AR-stimulated induction of lipolysis in isolated adipocytes from iWAT from these mice was almost completely abolished (Fig. 3d), suggesting that adipocyte lipolysis is required for in vivo induction of lipolysis via β-AR stimulation, as well as sucrose-induced potentiation of the lipolysis response to β-AR stimulation. These data also show that adipocyte lipolysis is necessary for sucrose-mediated hepatic steatosis (Fig. 3e). In adipose-specific ATGL knockout mice and their control genotype counterparts we observed significant effects of both genotype and sucrose intake on liver triglyceride content in both male (*p* = 0.004 and *p* = 7.72^−4^, respectively) and female mice (6.96^−4^ and *p* = 2.63^−6^, respectively). These effects were associated with an increase in liver triglyceride content in mice with the control genotype after sucrose intake relative to the non-sucrose group (males: 283.0%, *p* = 0.005; females: 457.6%, *p* = 1.0^−4^). We observed no difference in liver triglyceride content in knockout males after sucrose intake relative to knockout males that did not receive sucrose (*p* = 0.472), whereas males with the control genotype had 319.9% more triglycerides than knockout males after sucrose intake (*p* = 0.009). In contrast to our observations in male mice, female mice with adipose-specific ATGL knockout did demonstrate a difference in liver triglyceride content between the sucrose and non-sucrose groups (263.8%, *p* = 0.039), although this difference was not as robust at the effect of sucrose in female mice with the control genotype (457.6%, *p* = 1.0^−4^). Together with our observation that female mice with the control genotype acquired more liver triglyceride than knockout females after sucrose intake (181.6%, *p* = 0.016), this suggests an intermediate effect of ATGL knockout on liver triglyceride content in female mice. Thus, these data demonstrate that in male mice, adipocyte lipolysis is necessary for the development of hepatic steatosis following chronic liquid sucrose intake, whereas in female mice, impairment of adipocyte lipolysis attenuates but does not completely prevent hepatic triglyceride accumulation. These findings are in line with our earlier observation that in addition to increased NEFA re-esterification following sucrose intake, female mice have increased rates of de novo fatty acid synthesis, which would be expected to contribute to liver triglyceride content independently of NEFA derived from adipocyte lipolysis.

### Sucrose elicits distinct transcriptional responses in adipose tissue

**Fig. 4:**
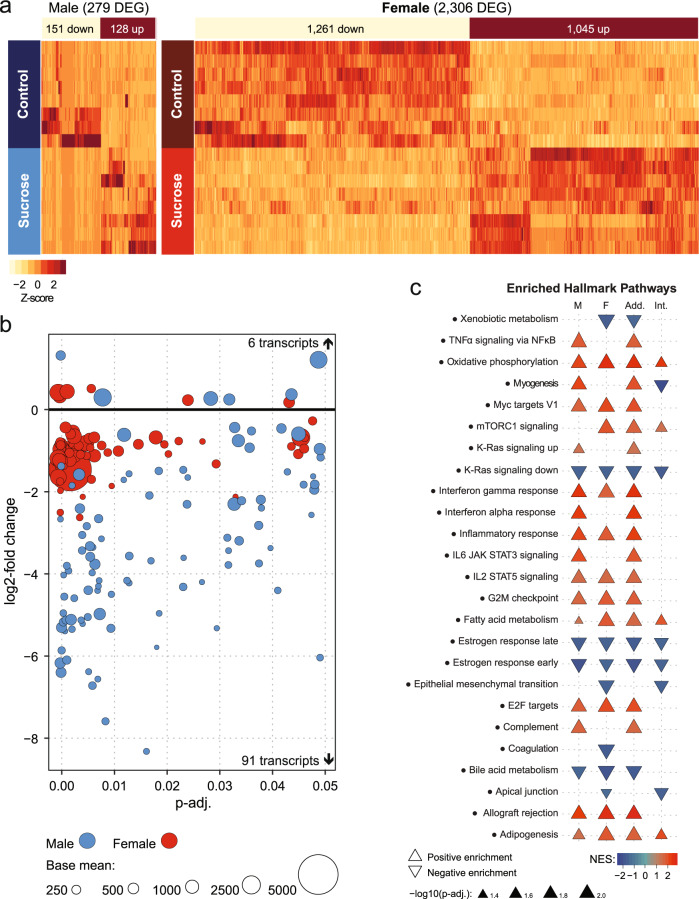
Sucrose intake elicits distinct transcriptional responses in adipose tissue from male and female mice. **a** Heat maps demonstrate a difference in the number of transcripts differentially expressed in inguinal adipose tissue following chronic liquid sucrose intake in each sex, as determined using negative binomial generalized models and Wald tests with Bonferroni correction in DESeq2 (MC: *n* = 8, MS: *n* = 8, FC: *n* = 8, FS: *n* = 8). **b** Bubble plot of the differentially expressed transcripts common to both male and female mice following sucrose intake demonstrating the log2-fold change (vertical spread), adjusted *p*-values (horizontal spread) and normalized count value (bubble size) for each sex as determined in **a**. **c** Top 25 gene sets enriched according to the Hallmark database. NES normalized enrichment score, M male, F female, Add additive effect of sex on the effect of sucrose intake, Int. sex sucrose interaction effect, MC Male Control, MS Male Sucrose, FC Female Control, FS Female Sucrose. Relative transcript expression, including Log2 fold change and adjusted *p*-values are available in Supplementary File 2, Gene Set Enrichment Analyses are available in Supplementary File 4, and raw data are available from the NCBI Gene Expression Omnibus, accession number GSE151358.

To gain insight into the adipose-specific mechanisms contributing to the divergent lipolysis responses we observed in adipocytes from male and female mice after chronic sucrose intake, we sought to identify how liquid sucrose consumption altered the adipose tissue transcriptome. Compared to their respective control groups, sucrose intake led to upregulation of 1045 gene transcripts in female mice and 128 gene transcripts in male mice (Fig. 4a and Supplementary Data File 2). Only six transcripts were commonly upregulated by sucrose intake in both sexes; these were: *Tma7*, *Ctsc*, *Rab21, Skap2, Tmf1,* and *Pla2g7* (Fig. 4b). We observed 1261 gene transcripts downregulated in adipose tissue from female mice after sucrose intake, whereas 151 transcripts were downregulated in male adipose tissue. Ninety-one transcripts were commonly downregulated in both male and female mice following sucrose intake (Fig. 4b).

To tease out any functional relationships between the differentially expressed genes common to both male and female mice, we performed string network analysis (Supplementary Fig. 4a). The only functional relationship identified for a protein encoded by one of the commonly upregulated transcripts was *Rab21*, which formed an interaction network with other Rab proteins encoded by downregulated transcripts: *Rab17* and *Rab25*. Several larger interaction networks were observed for proteins encoded by the transcripts commonly downregulated. These networks contributed to functional enrichment of pathways involved in tissue morphogenesis, cell proliferation and differentiation, as well as cell-cell adhesion and cell-cell communication (Supplementary Data File 3).

To identify which pathways and networks were altered by sucrose intake, including which pathways were also influenced by sex, we performed gene-set enrichment analyses comparing all differentially expressed genes we identified with the reference gene sets found in the Molecular Signatures Database (Supplementary Data File 4). We found 2048 pathways enriched in female mice following sucrose intake (1496 positively enriched, 552 negatively enriched), and 1378 pathways enriched in male mice (1168 positively enriched, 210 negatively enriched). An additive effect of sex on sucrose intake was observed for 2049 pathways (1650 positively enriched, 399 negatively enriched), whereas a sex:sucrose interaction effect was observed for 591 pathways (131 positively enriched, 460 negatively enriched). Among the positively enriched gene sets, those representing mitochondrial biogenesis, aerobic metabolism, and inflammation and immune responses were most commonly enriched in both sexes. Negatively enriched gene sets found in both sexes included those associated with cell proliferation and cancer, estrogen responsiveness, and bile acid metabolism. Additionally, male mice had positive enrichment of gene sets associated with myogenesis, muscle development and intracellular calcium dynamics, whereas female mice had negative enrichment of gene sets associated with amino acid metabolism and collagen biosynthesis. Sucrose intake and sex interacted to positively enrich gene sets associated with adipogenesis and mitochondrial metabolism, and negatively enrich gene sets associated with myogenesis and breast development. In almost all cases, female sex had an additive effect on enrichment of gene sets that were enriched in both sexes.

For the subset of gene sets in the Hallmark database (Fig. 4c and Supplementary Data File 4), sucrose intake led to enrichment of 65 distinct gene sets (45 positively enriched, 20 negatively enriched). In adipose tissue from male mice, sucrose enriched 16 Hallmark gene sets (13 positively enriched, three negatively enriched), whereas sucrose enriched 19 gene sets in adipose tissue from female mice (12 positively enriched, 7 negatively enriched). An additive effect of sex on sucrose intake was observed for 20 gene sets (16 positive, four negative), whereas ten gene sets showed sex:sucrose interactions (four positive, six negative). Positively enriched pathways include those associated with fatty acid metabolism and adipogenesis, immunoregulation, mitochondrial bioenergetics and the cell cycle, whereas negatively-enriched pathways include those associated with estrogen signaling, bile acid metabolism and cell-junction organization and cell-cell communication.

### Sucrose affects transcriptional regulation of adrenergic receptors

Since we were interested in identifying why male and female mice had divergent lipolysis adaptations in isolated adipocytes following sucrose intake, in addition to GSEA, we identified a subset of transcripts that encode either the enzymes and proteins that facilitate lipolysis (Supplementary Fig. 4b), or the receptors that regulate lipolysis activity either canonically (Supplementary Fig. 4c) or non-canonically (Supplementary Fig. 4d)^38^. Transcripts encoding the enzymes and proteins that facilitate lipolysis were expressed similarly across all groups, although sucrose intake did increase the expression of transcripts encoding lipid droplet proteins known to moderate lipolytic activity (*Plin2* and *Plin5*). Expression of the transcript encoding the most abundant mouse adipose tissue β-AR, *Adrb3*, was reduced in female mice following sucrose intake (−11.7%, padj = 0.013), as was the anti-lipolytic transcript *Adra2a* (−62.2%, padj = 0.041). A sex:sucrose interaction effect was observed for the β-AR desensitizer *Arrb1* (padj = 0.034), with female mice appearing to have reduced expression, although this did not attain statistical significance (−39.9%, padj = 0.202). These findings identify reduced transcriptional regulation of adrenergic signaling as a potential mechanism responsible for the attenuated stimulated lipolysis response we observed in adipocytes from female mice after chronic sucrose intake.

### Sex moderates serum bile acid concentrations

**Fig. 5:**
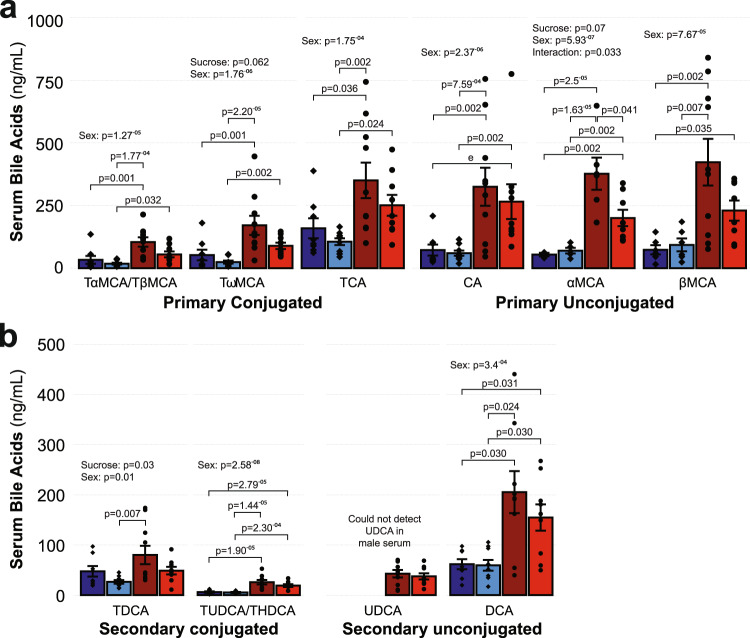
Composition of the circulating bile acid pool is influenced by both sex and sucrose intake. **a** Primary and **b** secondary conjugated and unconjugated bile acids in serum from male and female mice following chronic sucrose intake show sexual dimorphism as determined by two-way ANOVA with post hoc Tukey’s test (or Scheirer–Ray–Hare test and post-hoc Wilcoxon Rank Sum test with continuity correction for DCA, which demonstrated a non-parametric distribution). TαMCA/TβMCA: combined tauro-α-muricholic acid/tauro-β-muricholic acid (MC: *n* = 8, MS: *n* = 9, FC: *n* = 10, FS: *n* = 9), TωMCA: tauro-ω-mecholic acid (MC: *n* = 8, MS: *n* = 9, FC: *n* = 10, FS: *n* = 9), TCA taurocholic acid (MC: *n* = 8, MS: *n* = 9, FC: *n* = 10, FS: *n* = 9), CA cholic acid (MC: *n* = 8, MS: *n* = 9, FC: *n* = 10, FS: *n* = 9), αMCA α-muricholic acid (MC: *n* = 3, MS: *n* = 5, FC: *n* = 6, FS: *n* = 8), βMCA: β-muricholic acid (MC: *n* = 6, MS: *n* = 6, FC: *n* = 10, FS: *n* = 8), TDCA: tauro-deoxycholic acid (MC: *n* = 8, MS: *n* = 9, FC: *n* = 10, FS: *n* = 9), TUDCA/THDCA: combined tauro-ursodeoxycholic acid/tauro-hyodeoxycholic acid (MC: *n* = 8, MS: *n* = 9, FC: *n* = 9, FS: *n* = 9), UDCA: ursodeoxycholic acid (MC: *n* = 1, MS: *n* = 0, FC: *n* = 10, FS: *n* = 9), DCA deoxycholic acid (MC: *n* = 8, MS: *n* = 9, FC: *n* = 9 FS: *n* = 9). Data displayed are the group means ± standard error with biologically independent values overlayed (see figure for *p*-values). Source data are provided in the source data file.

Bile acids play a role in nutrient sensing and are emerging as key players in the regulation of both lipid and carbohydrate metabolism^38,39^. Additionally, bile acid signaling thorough Tgr5/*Gbar1* has been suggested as a potential non-canonical regulator of adipose tissue lipolysis^38^. Given that one of the gene sets negatively enriched in our transcriptomic data was bile acid signaling, we sought to characterize whether either sex or sucrose intake influenced the composition of the circulating bile acid pool. Sex was a moderating factor for all bile acids we measured (all *p* ≤ 0.01, Fig. 5a, b). Under control conditions, compared to males, female mice had increased concentrations of all unconjugated (Fig. 5a; cholic acid, CA, 348.5% *p* = 0.002; α-muricholic acid, αMCA, 592.1% *p* = 2.5^−5^; β-muricholic acid, βMCA, 475.1% *p* = 0.002) and conjugated primary bile acids measured (Fig. 5a; combined tauro-α-muricholic acid/tauro-β-muricholic acid, TαMCA/TβMCA, 213.7% *p* = 0.001; tauro-ω-mecholic acid, TωMCA, 225.0% *p* = 0.001; taurocholic acid, TCA, 120.1% *p* = 0.036), two unconjugated secondary bile acids (Fig. 5b; ursodeoxycholic acid, UDCA, detected only in serum from female mice; deoxycholic acid, DCA, 233.3% *p* = 0.03) and two conjugated secondary bile acids (combined tauro-ursodeoxycholic acid/tauro-hyodeoxycholic acid, TUDCA/THDCA, 339.7% *p* = 1.9^−5^). Similar findings were observed following sucrose intake, with female mice maintaining higher concentrations of CA (342.4% *p* = 0.002), αMCA (185.7% *p* = 0.002), TαMCA/TβMCA (205.1% *p* = 0.032), TωMCA (262.9% *p* = 0.002), TCA (137.1% *p* = 0.024), UDCA (not detected in male serum), DCA (160.8% *p* = 0.030), and TUDCA/THDCA (261.3% *p* = 2.3^−4^) compared to male mice that also received sucrose. An effect of sucrose intake was observed for TDCA (*p* = 0.033), whereas a sex:treatment interaction effect was observed for αMCA (*p* = 0.033), the latter reflected as a reduction in αMCA concentrations in female mice after sucrose intake compared to the female control group (−46.9%, *p* = 0.041).

### Sex modulates the effect of chronic sucrose intake on the microbiome

**Fig. 6:**
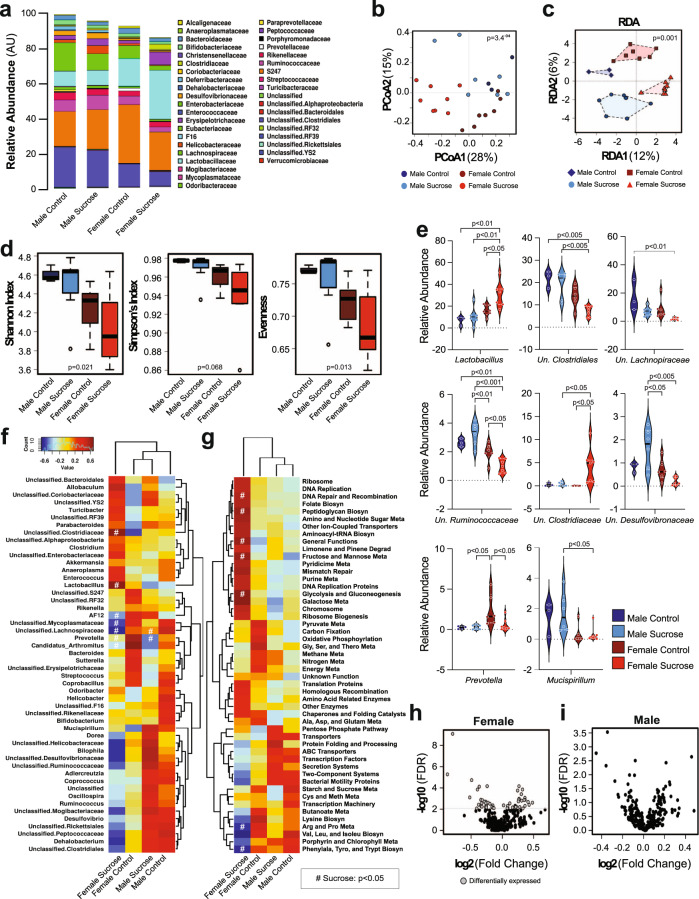
Sucrose intake has sex-specific effects on the microbiome. **a** Microbiome relative abundance at the family level. **b** Beta diversity assessed by Bray–Curtis analysis displayed as principal coordinate analysis (PCoA; PERMANOVA R2 0.33, *p* = 3.4^−4^; Anosim R = 0.512, *p* = 1.0^−4^; PERMDISP2 *p* = 0.355; MC: *n* = 3, MS: *n* = 7, FC: *n* = 7, FS: *n* = 8). **c** Redundancy analysis (RDA) identified significantly dissimilar group clustering of beta diversity (Variance = 117, F = 2.12, *p* = 0.001). **d** Female mice have reduced alpha diversity as determined by Shannon (*p* = 0.021), Simpson’s (*p* = 0.068), and Evenness indices (*p* = 0.013; MC: *n* = 3, MS: *n* = 7, FC: *n* = 7, FS: *n* = 8, boxes extend between the Q1 to Q3 interquartile range with the line representing the median and the whiskers extending between the minima and maxima. Dots represent statistical outliers). **e** Significant changes in the top 8 most abundant genera and unclassified (Un.) family levels as assessed by one-way ANOVA with post hoc Tukey’s tests (MC: *n* = 3, MS: *n* = 7, FC: *n* = 7, FS: *n* = 8; violin plots extend between the minima and maxima with the solid line representing the mean, the dotted lines representing the Q1 to Q3 interquartile range, and the width representing the distribution of data. Each biologically independent value is overlayed). **f** Heatmap of the 50 most abundant genus or unclassified family levels as calculated by spearman’s rank correlation coefficient. Significance was determined using one-way ANOVA assessing the effect of sucrose (^#^p < 0.05 compared to same-sex control group). **g** Heatmap of the 50 most abundant predicted metabolic pathways as determined by PICRUSt and calculated by spearman’s rank correlation coefficient. Significance was determined using one-way ANOVA assessing the effect of sucrose (^#^*p* < 0.05 compared to same-sex control group). **h**, **i** Deseq2 volcano plot of KEGG genes in PICRUSt from female (**h**), and male (**i**) mice following sucrose intake compared to their respective control groups. MC Male Control, MS Male Sucrose, FC Female Control, FS Female Sucrose. Source data are provided in the source data file, Log2 fold change and log10 FDR adjusted p-values are available in Supplementary File 5, and raw data are available from the NCBI BioProject database, accession number PRJNA883667.

Primary bile acids undergo deconjugation and other modifications as a result of the metabolic activities of gut microbiota^39^. Since sucrose intake had an effect on the composition of the serum bile acid pool in female mice, we next sought to determine whether dysbiosis of the gut microbiome might be associated with some of the sex- and/or treatment-specific differences we observed. At the phylum level, stool samples from female mice contained greater levels of Bacteroidetes than samples from male mice. At the family level, stool from females contained lower levels of unclassified *Rickettsiales* and *Clostridiales* and greater levels of *Lactobacillaceae*. Chronic sucrose intake increased the amount of *Lactobacillaceae* while decreasing *Prevotellaceae* (Fig. 6a). Beta diversity was assessed by Bray–Curtis and is shown as a principal coordinate analysis (PCoA; Fig. 6b), where PCoA 1 explained 28% of diversity and discriminated sucrose vs controls. Dissimilar clustering was observed between groups (PERMANOVA R2 0.33, *p* = 3.4^−4^; Anosim R = 0.512, *p* = 1.0^−4^) while dispersion variability across groups was not significantly different (PERMDISP2 *p* = 0.355). Redundancy analysis (RDA) identified groups as being significantly different (Variance = 117, F = 2.12, *p* = 0.001) (Fig. 6c). Alpha diversity was assessed by Shannon, Simpson’s and Evenness indices (Fig. 6d), where males showed greater diversity than females, and sucrose feeding decreased diversity in females, but not males. At the genus level, female mice that did not receive sucrose displayed increased *Lactobacillus*, but reduced levels of unclassified *Clostridiales* compared to male mice that did not receive sucrose. Sucrose intake increased *Lactobacillus* and unclassified *Clostridiaceae* in females, while decreasing unclassified *Lachnospiraceae*, unclassified *Mycoplasmataceae*, *Prevotella*, *Candidatus Arthromitis* (Segmented filamentous bacteria), and AF12 compared to female mice that did not receive sucrose. (Fig. 6e, f, Supplementary Data File 5). Predictive metagenomics showed multiple changes in metabolic pathways in female mice fed sucrose, while no differences were observed for male mice (Fig. 6g). Specifically, amino acid metabolism was markedly decreased while fructose and mannose metabolism as well as glycolysis and gluconeogenesis KEGG pathways were upregulated in females following chronic sucrose intake. These differences were driven by significant changes in 52 microbial genes after FDR correction, while no significant differences were seen in males (Fig. 6h, i, see also Supplementary Data File 5).

## Discussion

Here, we show that chronic intake of liquid sucrose at concentrations relevant to typical human consumption induces sexually dimorphic metabolic effects in liver, adipose tissue, and the microbiome; differences that likely contribute to the severity of sucrose-induced hepatic steatosis and the progression of NAFLD. We provide evidence that in the absence of systemic impairments in insulin responsiveness or glucose tolerance, sex is a moderating factor that interacts with the effects of high dietary sucrose intake to direct lipid storage and determine the contribution of de novo fatty acid synthesis to the hepatic triglyceride pool. These findings provide physiologic insight into how sex influences the regulation of adipose-liver crosstalk in response to dietary sugar intake and highlight the importance of extrahepatic metabolism in the pathogenesis of diet-induced steatosis and NAFLD.

Sucrose is a naturally occurring disaccharide comprised of equimolar amounts of the monosaccharides glucose and fructose. The refined form of sucrose, together with high-fructose corn syrup (which is not meaningfully different from sucrose in its composition), are the most commonly added sugars in the human diet^40,41^. The ability of the fructose monosaccharide to bypass glycolytic regulation and proceed directly toward de novo fatty acid synthesis has long been speculated as the mechanism responsible for initiating NAFLD in response to the intake of dietary sugars^24^. Indeed, there are many reports in both preclinical^15,42,43^, and human populations^29,44,45^ that support the assertion that fructose ingestion contributes to steatosis. However, fructose is rarely consumed in isolation, nor are added sugars consumed at the exaggerated concentrations frequently used to induce steatosis in animal models^26^, and there is evidence in humans that suggests only those with existing metabolic disease are subject to fructose-induced gains in hepatic triglyceride when consumed in energy balance^44,46–48^. This lack of translatability, along with the long-supported notion that hepatic metabolism is tightly coupled to both endocrine signaling and systemic cues from metabolic intermediates generated endogenously by other tissues^49^, makes it difficult to draw mechanistic conclusions about the initiating pathophysiology of NAFLD resulting from the intake of dietary sugars.

In the current study, we have used a model of chronic sugar intake where chow-fed mice received sucrose in their drinking water at a concentration representative of many sugar sweetened beverages made for human consumption^50^. This ‘dose’ of sucrose was effective at increasing adiposity and inducing hepatic steatosis without causing systemic disruptions to insulin responsiveness or glucose tolerance. Notably, in contrast to previous studies^51–53^, we observed that hepatic triglyceride accumulation following physiologically plausible sucrose intake was primarily driven by the re-esterification of fatty acids derived from adipocyte lipolysis in male mice, and by a combination of re-esterification and hepatic de novo fatty acid synthesis in female mice. Furthermore, we showed that ATGL-driven adipocyte lipolysis was required for sucrose-associated steatosis, although inhibition of lipolysis was only partially protective against steatosis in female mice. Together with our observation that males had a higher capacity to store lipid in their adipose tissue than females, these findings suggest that steatosis in the setting of high sugar intake may begin as a secondary consequence of dysregulated adipose tissue lipolysis and a reduced ability to store lipid in adipocytes, rather than a primary defect in hepatic metabolism per se. If this assertion is correct, it may help to explain why the most effective treatments for NAFLD to date have involved the reduction of adiposity, either via diet and exercise^54^ or following surgical weight loss^55^.

The preservation of systemic insulin responsiveness (and glucose tolerance) in our model demonstrate a clear decoupling of the mechanisms responsible for steatosis and those responsible for impairments in insulin sensitivity following chronic sucrose intake and, in the case of female mice, overt obesity. In humans, sugar sweetened beverage consumption is strongly associated with NAFLD^6–11^ but not always with obesity^56^. Additionally, NAFLD does not always present in parallel with obesity or insulin resistance in humans^5^ or in animal models^57–59^ suggesting that the etiology of NAFLD likely deviates from that of other metabolic comorbidities. Our findings suggest that in the case of NAFLD resulting from high dietary sucrose intake, steatosis development is unlikely to be intrinsically linked with mechanisms responsible for systemic insulin resistance. However, it is worth acknowledging that in the current study, mice receiving sucrose rapidly reduced their solid food intake, presumably as a way of regulating their total caloric intake. In one respect, the lack of difference in energy intake between mice receiving sucrose and those in the control groups strengthens some of our observations because it prevented the confounding effect of excess energy intake. On the other hand, the macronutrient intake of the mice receiving sucrose was altered beyond the addition of sucrose. Based on the macronutrient breakdown provided by the manufacturer of the chow diet, male and female mice receiving sucrose consumed less protein (14.2% and 16.9%, respectively, compared to 25%) and less fat (9.6% and 11.5%, respectively, compared to 17%), and these differences may have contributed to some of the characteristics we observed in mice following chronic sucrose intake—including the preservation of insulin sensitivity. For example, lower intake of protein and the subsequent reduction in branched chain amino acid availability may have contributed to the maintenance of systemic insulin sensitivity and glucose tolerance in mice receiving sucrose^57–61^.

Our observation that male and female mice had mechanistically different responses to sucrose intake was not surprising. In silico modeling suggests NAFLD develops through distinct metabolic processes in males and females^35^, whereas epidemiological data strongly support an increased prevalence of NAFLD in men^32,33^, yet worsened NAFLD severity in women^32^. Indeed, our observation that the severity of sucrose-induced steatosis was greatest in female mice is in line with previous studies that report worsened steatosis in female rats following high fructose^62^, or high fat, high fructose diets^63^. Together with the observation that women increase de novo fatty acid synthesis in response to acute fructose ingestion whereas men do not^64^, our findings add physiologic insight as to why females develop more severe steatosis than males in response to high dietary sugar intake.

In addition to highlighting the importance of metabolic crosstalk between liver and adipose tissue in the development of NAFLD, our study has identified several distinct transcriptional regulatory pathways that are induced in adipose tissue by chronic liquid sucrose intake. Many of the pathways we identified were enriched in both male and female mice, whereas many gene sets also demonstrated an additive effect of sex. For example, we observed marked positive enrichment of gene sets associated with mitochondrial biogenesis and oxidative phosphorylation, an effect that was augmented in adipose tissue from female mice. Interestingly, two of the negatively enriched gene sets we observed included genes associated with estrogen responsiveness and bile acid signaling, two pathways with well-established links to lipid metabolism. In the present study we were able to link some of the sexual dimorphism we observed back to differences in bile acid availability, likely due to differences in the microbiome. And although we saw down-regulation of estrogen signaling in adipose tissue, reductions in the transcript encoding Estrogen Receptor α (ERα; *Esr1*) were only observed in adipose tissue from female mice (Supplementary Fig. 4e). Although further investigation of how sucrose intake lowers estrogen signaling is beyond the scope of the current work, we can speculate as to how loss of estrogen signaling contributes to the divergent phenotypes we observed. Estradiol interacting with ERα is a negative regulator of adipocyte hypertrophy^65,66^. Thus, it is plausible that estrogen signaling becomes downregulated in adipose tissue as a means to increase adipocyte lipid storage in response to sucrose intake. In liver, estradiol has been shown to recruit ERα to the lipogenic genes *Fasn* and *Acaca*, resulting in their transcriptional inhibition^67^. Should this function translate across tissues, it is possible that lower endogenous concentrations of circulating estradiol in combination with attenuated basal expression of adipose tissue *Esr1* would assist male mice to rapidly increase their adiposity in response to sucrose intake, whereas the need to attenuate a higher basal *Esr1* expression would likely contribute to the milder adiposity gains we observe in female mice. Additionally, abrogation of circulating estradiol via ovariectomy^66,68^, or selective loss of ERα^67,69^ in liver each result in steatosis, with the latter showing sexual dimorphism in the steatosis response^69^. Reductions in estrogen signaling in response to sucrose intake would be expected to increase hepatic de novo fatty acid synthesis via upregulation of *Fasn* and *Acaca*^67^. In support of this notion, liquid fructose ingestion (13% w/v) has been shown to cause a reduction in hepatic ERα protein expression concomitant with increases in *Fasn, Acaca*, and steatosis^70^, whereas de novo fatty acid synthesis induced by sucrose, fructose or glucose has been shown to reduce the hepatocyte-produced sex hormone binding globulin (SHBG)^71^, which is thought to interact with ERα to regulate the transport of estrogens (and testosterone) into tissues in humans^72^. In the current study, hepatic expression of *Esr1* was reduced in female mice following sucrose intake yet remained unaffected in males (Supplementary Fig. 4f). Future studies will determine the mechanisms responsible for the reductions in hepatic and adipose *Esr1*/ERα we observe in females following chronic sucrose intake^70–72^.

**Fig. 7:**
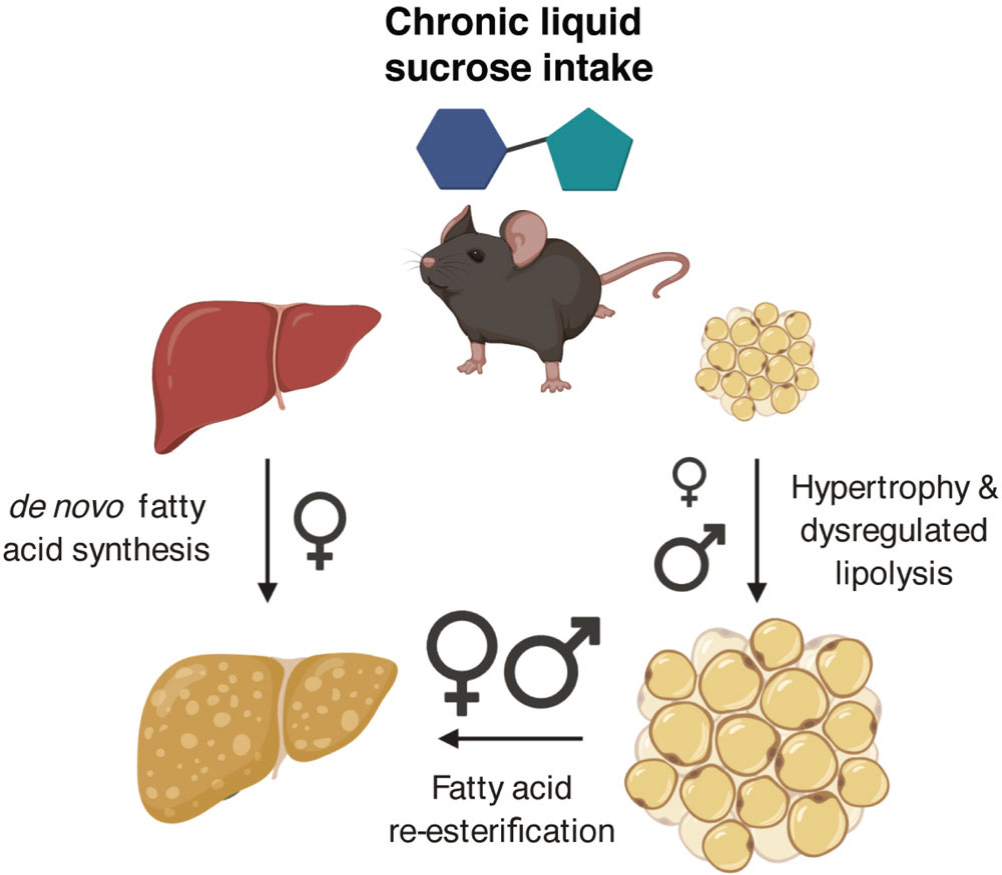
Summary of main findings. Chronic liquid sucrose intake at physiologically relevant concentrations has sexually dimorphic effects on liver and adipose tissue, differences that likely contribute to the severity of the resultant steatosis. In this study, we observed that male mice were able to convert excess dietary sucrose into fatty acids that were readily stored as triglyceride in the adipose tissue, whereas female mice were unable to expand their adipose tissue as effectively as males, leading to additional manufacturing (and storage) of new fatty acids in the liver. Our results suggest that liquid sucrose intake induces steatosis mainly via ‘spillover’ of fatty acids from dysregulated adipocyte lipolysis in both sexes, with additional contributions from de novo fatty acid synthesis occurring in females. Future studies will identify the mechanisms responsible for these divergent responses and allow us to better target therapeutic strategies for the treatment of NAFLD. Image created with BioRender.com.

In conclusion, we have shown that sex is a moderating factor in the regulation of lipid metabolism in both adipose tissue and liver. We demonstrate that mice consuming liquid sucrose at concentrations relevant to human consumption develop steatosis primarily as a result of dysregulated adipose tissue lipolysis and that the increased severity at which female mice develop steatosis is driven by upregulation of de novo fatty acid synthesis in addition to enhanced re-esterification (Fig. 7). We speculate that the sexually dimorphic metabolic responses in adipose tissue and liver are likely due to impaired estrogen signaling either directly, through ERα, and/or indirectly, via a circulating intermediate acting on ERα. Future studies will investigate the mechanisms by which chronic sugar intake attenuates estrogen signaling and its role in liver-adipose crosstalk. In addition, our findings highlight the importance of preclinical studies that include both sexes in the experimental design, and the need for increased translatability in relation to preclinical research that involves the study of macronutrient metabolism. Lastly, when considering treatment strategies for reversing NAFLD, greater emphasis should be placed on extrahepatic lipid metabolism and the influence of sex and sex hormone signaling.

## Methods

### Generation of mice and experimental design

Female mice homozygous for a LoxP-modified *Pnpla2* allele (B6N.129S-*Pnpla2*^tm1Eek/J^, stock number 024278^36^; *Pnpla2*^Fl/Fl^) and male mice hemizygous for an adipose tissue-specific Cre recombinase, under control of the adiponectin promotor (B6FVB-Tg^(*Adipoq*-cre)1Evdr/J^, stock number 028020^37^; Cre/^+^) mice were purchased from The Jackson Laboratory and crossed to generate breeding stock that were heterozygous for the targeted mutation of *Pnpla2*, with or without hemizygosity for the *Adipoq*-cre transgene (Fl/+;Cre/+ or Fl/+;+/+). The originating mice in the floxed strain were congenic to a C57BL/6NJ background, whereas the mice in the Cre strain were congenic to a C57BL6/J background. To generate experimental mice with or without adipose-specific deletion of exons 2-7 of *Pnpla2*, Fl/+;Cre/^+^ mice were mated with Fl/+;+/+ mice. Four of the resulting six genotypes were utilized in this study; the wild-type mice, with no genetic alterations (+/+;+/+), mice with adipose-specific deletion of *Pnpla2*, (Fl/Fl;Cre/^+^), and two additional control groups (Fl/Fl;+/+ and + /+;Cre/^+^). All mice were of a single generation and were genotyped at 14 days of age by PCR of tail DNA. Following genotyping, mice were randomly assigned to receive either water (control groups) or sucrose (10% w/v, prepared in distilled water) in their drinking water once they reached experimental age. Both male and female mice were weaned onto standard rodent chow (Envigo 7912) and water at 21–24 days of age, with their assigned experimental treatment beginning at 10 weeks of age. Mice were housed in ventilated cages with corn cob bedding in a humidity and temperature-controlled environment at ~22 °C with free access to food and water (or water containing 10% w/v sucrose). This concentration of sucrose was chosen because it closely approximates the concentration of sugar found across many readily available sugar-sweetened beverages made for human consumption^50^. The bulk of experiments were performed on mice of the + /+;+/+ genotype. In instances where Fl/Fl;Cre/^+^ mice were studied alongside all three control groups, the control group data (when not different between groups) were combined into a single group and labeled Control. Due to increased fluid intake of mice receiving sucrose, all cages were changed twice weekly. For all experiments, both male and female mice were studied. All procedures were performed in accordance with the National Institute of Health Guidelines for the Care and Use of Experimental animals and were pre-approved by the University of Tennessee Health Science Center Institutional Animal Care and Use Committee (protocol #3180).

### Longitudinal body composition monitoring

Mice were weighed and body composition was non-invasively measured using an EchoMRI 1100 (EchoMRI), beginning at 4 weeks of age, and was measured every week thereafter for the duration of the study.

### Indirect calorimetry, physical activity monitoring and measurement of food and water intake

At 20 weeks of age, mice were individually housed in a home cage-style Comprehensive Laboratory Animal Monitoring System (CLAMS, Columbus Instruments), for two weeks (one week at 25 °C, 1 week at 28 °C). Following a 24 h acclimation period, VO_2_ and VCO_2_ were measured via open-circuit indirect calorimetry using Oxymax software (v5.24 Columbus Instruments). Energy expenditure was calculated using the Lusk equation^73^. Total activity was calculated as the combined number of infrared beam breaks along the X- and Y-axes, whereas ambulatory activity was calculated as the combined number of consecutive X- and Y-axes beam breaks occurring in a single series. Food intake from suspended feeder baskets was determined via a linked load cell. Water/sucrose intake was measured manually, by regular weighing of the water bottles. Rates of fat and carbohydrate oxidation were calculated from indirect calorimetry data as previously described, assuming negligible protein oxidation^74^. Both body fat and lean mass were included as covariates in the models used to analyze data obtained from the CLAMS experiments.

### Glucose and insulin tolerance tests

Glucose and insulin tolerance were assessed at 24 weeks of age, with at least 4 days between tests. Mice were fasted for 6 h starting at the beginning of the photophase. Fasting glucose was measured via tail cut using a hand-held glucometer (OneTouch Ultra2, LifeScan IP Holdings) with GenUltimate test strips (PharmaTech Solutions, Inc.). Mice received an intraperitoneal injection of either D-glucose (2 g/kg lean mass) or insulin (1.0 U/kg lean mass; Humulin R-500, Lilly) in PBS (for glucose and insulin tolerance tests, respectively), and the change in blood glucose concentration was monitored every 15 min for 2 h.

### In vivo induction of lipolysis

At 22 weeks of age, mice were anesthetized with isofluorane and a blood sample was drawn via heparinized capillary from the retroorbital sinus. After a short recovery period, mice were injected intraperitoneally with 10 mg/kg isoproterenol (prepared in saline; I6504, Sigma Aldrich). A second blood sample was collected from anesthetized mice 15 min post-injection. Blood was allowed to clot over ice before being centrifuged at 500 g for 20 min at 4 °C. Glycerol and NEFA concentrations in serum were determined colorimetrically, using commercially available reagents (F6428, Sigma Aldrich; NEFA-HR(2), Wako Life Sciences, Inc.).

### Tissue collection

At 24 weeks of age mice were given a single intraperitoneal bolus of deuterium oxide (151882, Sigma Aldrich) containing 0.9% NaCl, at a final enrichment of 4.5 ± 0.54% of total body water immediately prior to the light phase (ZT-0.5). All water sources, including sucrose drinking water, were removed from the cages at the time of injection. Seven hours later, mice were anesthetized with isofluorane and blood was drawn from the retroorbital sinus and allowed to clot over ice. An unclotted aliquot was snap frozen in liquid N_2_ for later determination of deuterium body water enrichment. Mice were then euthanized by cervical dislocation and tissues harvested. The left lateral lobe of the liver, right gonadal adipose depot (gWAT) and right dorsolumbar-inguinal adipose depot (iWAT) were immediately freeze-clamped between stainless steel paddles pre-cooled in liquid N_2_ and stored at −80 °C for later analyses. The left iWAT depot was dissected out whole, quickly weighed, and placed into Krebs-Ringer-Bicarbonate-HEPES buffer (KRBH) for subsequent collagenase digestion and isolation of primary adipocytes. A portion of the median lobe of the liver was fixed in 10% neutral buffered formalin for 24 h before being dehydrated in ethanol and imbedded for histology.

### Preparation of adipocytes and ex vivo lipolysis

Primary adipocytes were isolated from iWAT. Briefly, adipose tissue was allowed to recover in KRBH buffer containing 2.5% BSA for 20 min before being chopped into tiny pieces and incubated in KRBH buffer containing 0.5% BSA and 1% collagenase (type II; 17101015, Gibco) for 1 h in a 37 °C shaking water bath. Primary adipocytes were separated from the stromal vascular fraction via a series of low centrifugation wash steps using KRBH containing 2.5% BSA. Aliquots of packed adipocytes were incubated in KRBH buffer with or without [100 μM] isoproterenol for 1 h at 37 °C. Buffer infranatants were collected, heat inactivated for 10 min at 85 °C, and analyzed for glycerol content using a commercially available kit (F6428, Sigma Aldrich). Adipocyte number was determined using a hemocytometer and cell counts were used to normalize rates of lipolysis to the number of cells present in the incubation medium.

### Tissue metabolite determination

Blood glucose concentrations were measured in whole-blood using a hand-held glucometer (OneTouch Ultra2, LifeScan IP Holdings) with GenUltimate test strips (PharmaTech Solutions, Inc.). Serum was separated from blood via centrifugation at 500 g for 20 min at 4 °C. Serum insulin and corticosterone concentrations were measured using ELISA’s (90080 and 80556, Crystal Chemical). Liver triglycerides were extracted via chloroform-methanol (2:1) extraction, evaporated overnight, re-suspended in butanol-methanol-Triton-X114 and measured colorimetrically (TR0100, Sigma Aldrich).

### De novo fatty acid and triglyceride synthesis

Deuterium oxide rapidly mixes with body water, allowing H^2^ to be incorporated into biosynthetic pathways that use H_2_O. Newly synthesized products can be quantified by measuring the H^2^ label present in the total pool of the product of interest. In this study, mice were provided a single bolus of 0.9% saline prepared with deuterium oxide 7 h before tissue collection and all other water sources were removed so as not to dilute the label. This short labeling period (hours rather than days) was chosen in order to minimize the contribution of recycled label (from product metabolism) into triglyceride-bound palmitate (which reflects the contribution of de novo fatty acid synthesis to the total triglyceride pool) and glycerol (which reflects the rate of triglyceride esterification)^75–77^.

Total triglycerides were isolated from liver samples via chemical hydrolysis and extraction^76^. Samples were dried down, derivatized, and converted to their trimethylsilyl derivatives for the measurement of glycerol and fatty acids (palmitate, oleate, and stearate) after the addition of internal standards^77^. Isotope enrichments were determined by gas chromatography (GC)–mass spectrometry (MS) using chemical ionization. Deuterium labeling of body water was determined in whole-blood samples by acetone exchange^77^. All MS analyses were carried out on an Agilent 5977B MSD (CI or EI mode) coupled to a 7890B GC. The contribution of newly synthesized glycerol and fatty acids to the total triglyceride pool was calculated as previously described^75–77^.

### Serum bile acid determination

Serum was prepared and bile acids were separated and quantified by LC-MS as previously described in ref. 78. Briefly, serum samples were combined with methanol, vortexed, and incubated for 20 min at room temperature before being centrifuged. The supernatant was transferred to new tubes and evaporated under N_2_ before being resuspended in H_2_O (pH 3.0), purified on C18 SPE columns, and eluted in methanol. Samples and standards were injected into an Agilent 6460 Triple Quad LCMS/MS with 1290 UHPLC. Bile acids were separated on a Zorbax eclipse XDB C18 column (Agilent Technologies) using a mixture of15 mmol/L ammonium acetate (pH 5.3) and methanol (65:35 vol/vol).

### qPCR and trancriptomics

RNA was extracted from liver and iWAT using TRI Reagent (AM9738, Ambion) and column purified (12183025 Purelink RNA mini kit, Life Technologies). RNA was either quantified spectrophotometrically (liver RNA; Nanodrop 2000, ThermoFisher Scientific) or by Agilent 2100 Bioanalyzer (iWAT RNA). cDNA was synthesized from purified liver RNA using a High-Capacity cDNA Reverse Transcription Kit (4368813, Applied Biosystems). iWAT RNA was submitted to Novogene for cDNA library preparation and subsequent RNAseq analysis. For qPCR, liver cDNA was combined with the appropriate working quantitative PCR master mix, containing Power SYBR Green (4368708, Applied Biosystems) and the relevant primer pair (final concentration [100 nM] each; Integrated DNA Technologies). PCR conditions included an activation cycle of 95 °C for 10 min followed by 45 amplification cycles of 15 s at 95 °C, 15 s at 60 °C, and 10 s at 73 °C. CT values were quantified on QuantStudio 6 Flex Real-Time PCR System (Applied Biosystems). The ΔΔCT method was used to calculate the expression levels of mRNA transcripts using *Actb* as the housekeeping transcript. The expression of *Actb* in our liver samples was determined to be unaffected by sex or sucrose intake after being compared alongside other commonly used qPCR housekeeping transcripts, including *Rps18, Rpl13a*, *Rplp0, B2m*, and *Gapdh*. Data were expressed relative to the female control group. Statistical comparisons were made on log-transformed values to address the heteroscedasticity observed for many of the measured transcripts. The sequences of each of the primers used can be found in Supplementary File 1. For RNAseq, iWAT RNA samples were submitted to Novogene where mRNA was purified from total RNA using poly-T oligo-attached magnetic beads. After random fragmentation, NEB cDNA libraries were constructed, assessed for quality, and sequenced on an Illumina platform (paired-end 150 bp). Downstream analysis was performed using a combination of programs including STAR, HTseq, Cufflink, and Novogene’s wrapped scripts. Alignments were parsed using Tophat. Reference genome and gene model annotation files were downloaded from genome website browser (NCBI/UCSC/Ensembl) directly. Paired-end clean reads were aligned to the reference genome using STAR (v2.5). HTSeq v0.6.1 was used to count the read numbers mapped to each gene and the Fragments Per Kilobase of transcript per Million mapped reads (FPKM) of each gene was calculated based on the length of the gene and the mapped read counts.

### Differential gene expression and gene set enrichment analyses

FPKM data were normalized and compared in R Studio using the package DESeq2 v1.24.0^79^. Differential expression analyses were conducted to determine the effects of sucrose intake within each sex, the effect of sucrose intake with sex as a moderating effect, and to determine any sex:sucrose interactions. P-values were adjusted for multiple comparisons according to Benjamini and Hochberg^80^, and transcripts with an adjusted *p* < 0.05 were considered differentially expressed. String network analysis was performed on differentially expressed genes common to both male and female mice using string-db.org version 11.0^81^. Gene Set Enrichment Analysis (GSEA) of all differentially expressed genes was completed in R Studio using the package fgsea v1.10.1^82^. GSEA was completed with all gene sets in the Molecular Signatures Database reference gene set (MSigDB.v7.0), downloaded from https://www.gsea-msigdb.org/. A gene set was considered enriched when the nominal *p*-value was <0.01, the false discovery rate was <0.25, and the normalized enrichment score was >1.5 (for gene sets positively enriched) or less than −1.5 (for gene sets negatively enriched).

### Western blotting

Homogenates were prepared from ∼50 mg of frozen liver in RIPA buffer [Tris basic (50 mM), sodium deoxycholate (0.25%), NP-40 (1%), NaCl (150 mM), EDTA (1 mM), Na3VO4 (100 μM), NaF (5 mM), sodium pyrophosphate (10 mM), protease inhibitor cocktail] using stainless-steel beads and a Qiagen Tissue Lyser (30 Hz for 5 min). Homogenates were centrifuged at 4 °C for 10 min at 14,000 g, after which the protein concentration of supernatants was determined by BCA assay. Lysates of equal protein concentration were prepared in 2× Laemmli buffer containing 2-mercaptoethanol and heated at 95 °C for 5 min. Proteins were separated by SDS-PAGE and transferred to nitrocellulose membranes for Western blotting. Membranes were visualized for total protein using stain-free technology (BioRad) before being blocked in BSA for 1 h and incubated at 4 °C overnight in the relevant primary antibody. Blots were visualized after a 1-h incubation with infrared anti-mouse or anti-rabbit secondary antibody, using a LI-COR Odyssey fluorescent Western blotting system or a BioRad ChemiDoc. Protein expression was quantified using densitometry (Image Studio Lite; LI-COR) and normalized to total protein. β-Tubulin was included as a visual loading control for figures. Antibodies used include: SREBP-1c IgG-2A4 (557036, BD Pharmingen, 1:1000), FAS C20G5 (3180 Cell Signaling Technology, 1:1000), ACC1/2 C83B10 (3676, Cell Signaling Technology, 1:1000), phospho-ACC1/2 Ser79 (3661, Cell Signaling Technology, 1:1000), ChREBP (NB400-135, Novus Biologicals, 1:1000), PKLR (AV41699, Sigma-Aldrich, 1:200), β-Tubulin BT7R (MA5-16308, Invitrogen, 1:2000), Goat anti-Rabbit IgG Alexa Fluor 680 secondary antibody (A21109, 1:15,000, Invitrogen) and Donkey anti-Mouse IgG Alexa Fluor 790 secondary antibody (A11371, 1:15,000, Invitrogen). Full blots are available with the Source Data.

### Histology

Tissue samples were fixed in 10% neutral buffered formalin overnight, dehydrated in ethanol, cleared in Citrisolv, and embedded in Paraplast plus. Sections were cut to 10 µm and stained with hematoxylin and eosin. Images were obtained at a 40X objective (EVOS XL CORE, ThermoFisher Scientific).

### Microbiome analysis with illumina MiSeq sequencing and bioinformatics

Stool samples were collected fresh and snap frozen. Samples were resuspended in 500 μL of TNES buffer containing 20 μg of proteinase K and 150 μL of 0.1 zirconia beads. Following mechanical disruption using ultra-high-speed bead beating samples were incubated overnight at 55 °C with agitation. Total DNA was extracted using phenol chloroform isoamyl alcohol, and total DNA concentration per mg stool was determined by qRT-PCR. Purified DNA samples were sent to the Argonne National Laboratory (Lemont, IL) for amplicon sequencing using the NextGen Illumina MiSeq platform. Blank samples passed through the entire collection, extraction and amplification process remained free of DNA amplification.

Sequencing data were processed and analyzed using QIIME (Quantitative Insights into Microbial Ecology) v1.9.1. Sequences were first demultiplexed, then denoised and clustered into sequence variants. For bacteria, we rarified to a depth of 10,000 sequences. Representative bacterial sequences were aligned via PyNAST, taxonomy assigned using the RDP Classifier against GreenGenes (v13.8). Processed data were then imported into Calypso (v8.84) for further analysis and data visualization^83^. For PICRUSt, biom tables were filtered against GreenGenes databases, normalized, and sequences used to predict KEGG orthologs and pathway enrichments. Alpha diversity was determined via calculation of the Shannon Index, Simpson Index, and Evenness at a depth of 10,000 OTUs^84,85^. Bray–Curtis analysis was used to quantify beta diversity (intra-sample) and displayed as principal coordinate analysis (PCoA)^86^. Comparisons were made using PERMANOVA, Anosim, and PERMDISP2^83^. Differences in taxonomic relative abundance were determined by one-way ANOVA adjusted using the Bonferroni correction and false discovery rate (FDR) for multiple comparisons followed by Tukey’s test. Linear discriminant analysis of effect size (LEfSe) with nonparametric factorial Kruskal–Wallis sum-ranking was used to identify significantly abundant taxa, followed by unpaired Wilcoxon rank-rum testing to determine LDA scores. LDA scores greater than 2 were considered significant^87^. Deseq2 was used to calculate differentially expressed taxa between experimental groups in both sexes. Network analysis was generated from Spearman’s correlations, where pairwise correlations are used to ordinate nodes in a two-dimensional plot by PCoA and positive correlations with FDR-adjusted, *p* < 0.05 were presented as an edge^83^.

### Statistics

All statistical analyses were performed using R (v4.1.1) running in R Studio (build 492). Data with multiple repeated measures were analyzed by linear mixed effects modeling with likelihood ratio tests using the package lme4^88^. All other data were assessed for homoscedasticity and normality using Levene’s tests (package: car^89^) and Shapiro–Wilk tests, respectively. Any heteroscedastic data sets were log-transformed to attain homoscedasticity and re-assessed for normality. Normal data were analyzed using two-way ANOVA with interaction models and Tukey’s post-hoc analysis. Any two-way factorial non-normal data were analyzed using Scheirer–Ray–Hare tests (package: rcompanion^90^), with either Wilcoxon Rank Sum test adjusted for multiple comparisons using the Benjamini and Hochburg correction or Dunn analysis with Benjamini and Hochberg correction for multiple comparisons (package: FSA^91^). Data processing and visualization were completed using the packages Tidyverse^92^ and lubridate^93^. Significance was set a priori with a *p* < 0.05 considered significant.

### Reporting summary

Further information on research design is available in the Nature Research Reporting Summary linked to this article.

## Supplementary information

Supplementary Information

Description of Additional Supplementary Files

Supplementary Data 1

Supplementary Data 2

Supplementary Data 3

Supplementary Data 4

Supplementary Data 5

Reporting Summary

## Data Availability

Gene set enrichment analyses performed for this work relied on the publicly available Molecular Signatures Database reference gene sets (MSigDB.v7.0), available from https://www.gsea-msigdb.org/. Microbiome data generated for this project are available from the NCBI BioProject database, accession number PRJNA883667. RNAseq data generated for this project are available from the NCBI Gene Expression Omnibus, accession number GSE151358. Other data generated for this work are provided in the Source Data file. Source data are provided with this paper.

## Source data

Source Data
