# Supplementary material for: Chronic intake of high dietary sucrose induces sexually dimorphic metabolic adaptations in mouse liver and adipose tissue
Source: Nat Commun. 2022 Oct 13;13:6062. doi: 10.1038/s41467-022-33840-6 (PMC9561177; doi:10.1038/s41467-022-33840-6)
Supplement: Supplementary file 1 — Supplementary Information [file 41467_2022_33840_MOESM1_ESM.pdf]

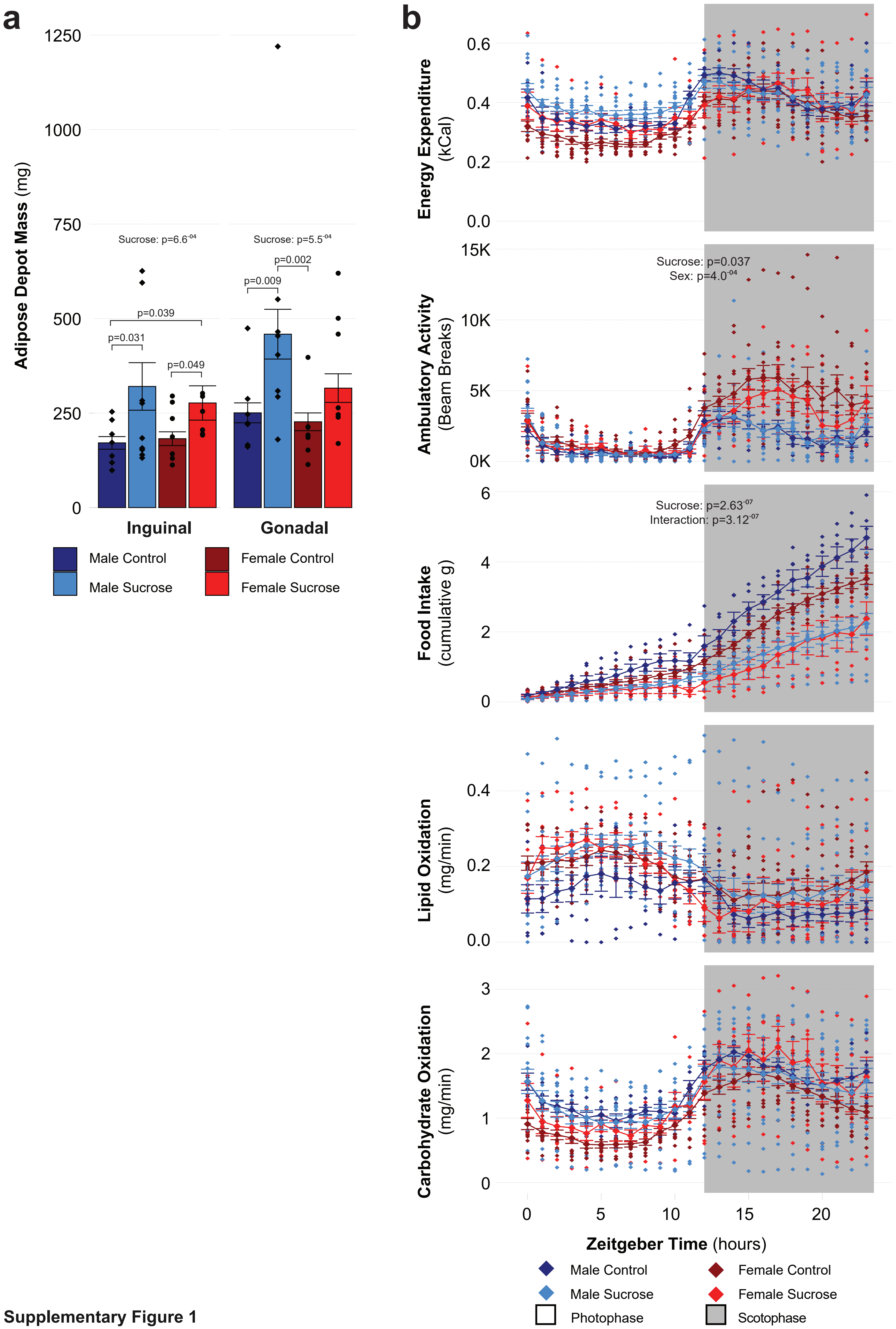

Supplementary Figure 1

Supplementary figure 1

Sucrose intake increases adiposity

a Sucrose intake increases the size of the inguinal and gonadal adipose depots as determined by Scheirer-Ray-Hare test and post hoc Dunn analysis with Benjamini and Hochberg correction for multiple comparisons (MC: n=14, MS: n=16, FC: n=14, FS: n=12; see figure for p-values). **b** 24-hour energy expenditure, ambulatory activity, cumulative food intake, lipid oxidation, and carbohydrate oxidation under thermoneutral conditions as determined by linear mixed effects models with likelihood ratio tests (MC: n=7, MS: n=14, FC: n=12, FS: n=9; see figure for p-values). Sucrose continues to reduce activity and food intake but no longer has an effect on energy expenditure or substrate oxidation. Data displayed are the group means ± standard error with biologically independent values overlayed. MC = Male Control, MS = Male Sucrose, FC = Female Control, FS = Female Sucrose. Source data are provided in the source data file.

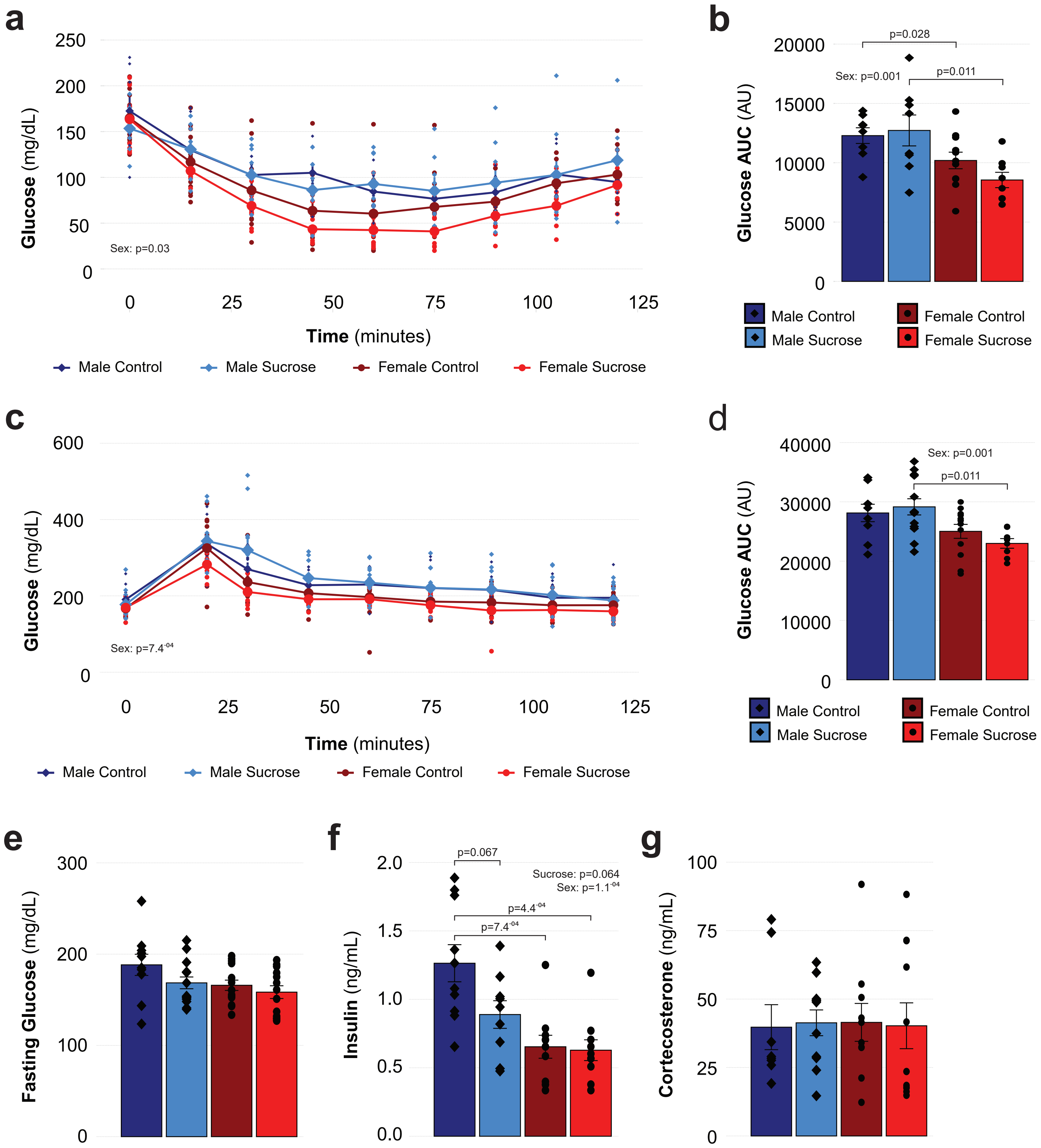

Supplementary Figure 2

Supplementary figure 2

Sucrose intake did not impair insulin responsiveness or glucose tolerance

a-b Neither insulin responsiveness (MC: n=8, MS: n=8-9, FC: n=11-14, FS: n=8-11; see figure for p-values) or **c-d** glucose tolerance (MC: n=9, MS: n=13, FC: n=12, FS: n=8; see figure for p-values) were affected by sucrose intake as determined by linear mixed effects models with likelihood ratio tests (temporal data; **a** and **c**) and two-way ANOVA with post hoc Tukey's tests (area under the curve; **b** and **d**). **e** Fasting blood glucose was not different between groups (MC: n=10, MS: n=14, FC: n=14, FS: n=13), **f** although an effect of sex was observed for fasting insulin concentrations as determined by two-way ANOVA with post hoc Tukey's test ($p=1.1^{-4}$; MC: n=10, MS: n=9, FC: n=10, FS: n=10). **g** Corticosterone concentrations were similar between groups (MC: n=8, MS: n=11, FC: n=10, FS: n=11). Data displayed are the group means ± standard error with biologically independent values overlayed. MC = Male Control, MS = Male Sucrose, FC = Female Control, FS = Female Sucrose. Source data are provided in the source data file.

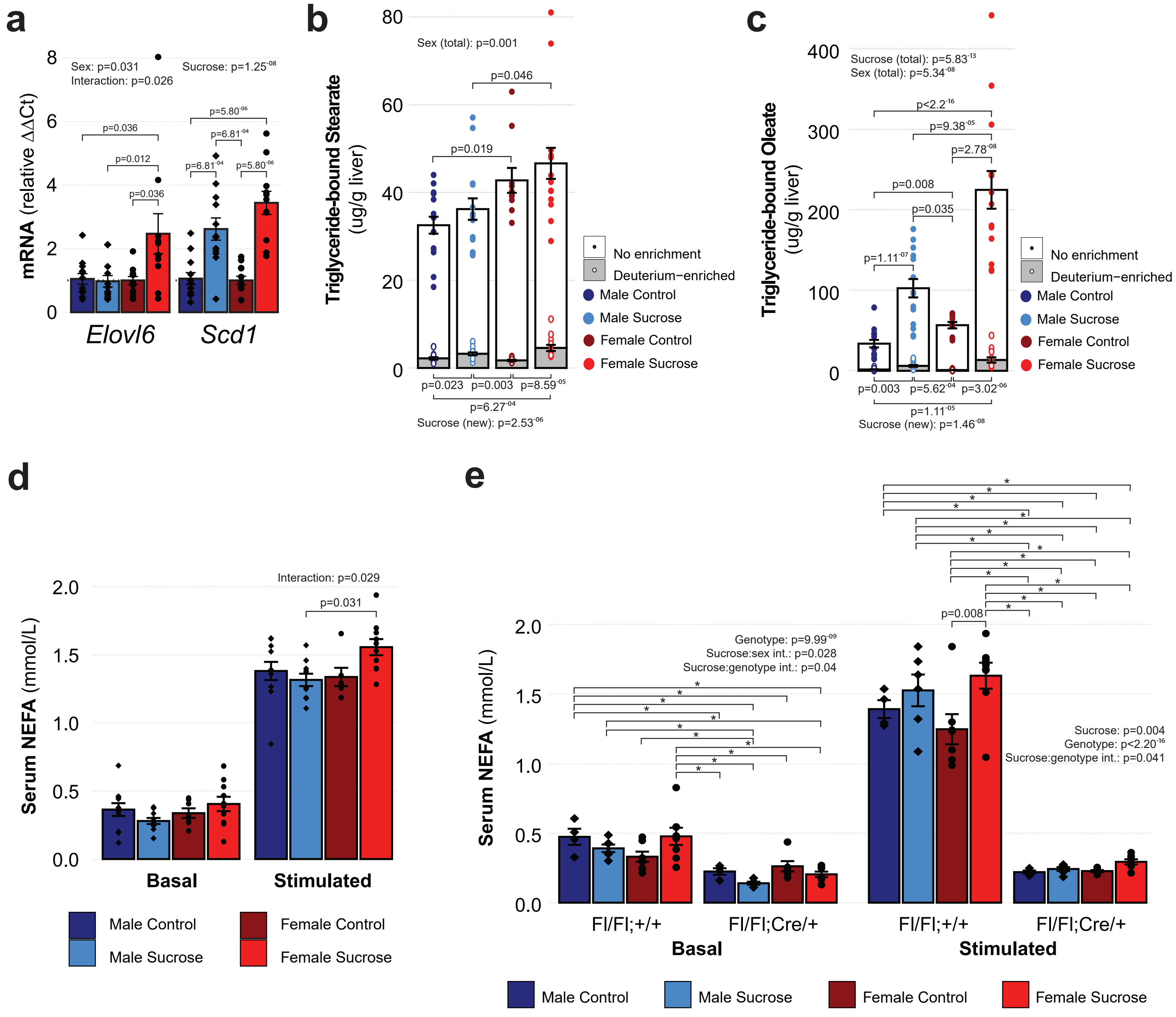

Supplementary Figure 3

Supplementary figure 3

Sucrose intake results in sex-dependent triglyceride modifications

a Transcriptional regulation of fatty acid modifications exhibited sex-specific sucrose effects as determined by two-way ANOVA with post hoc Tukey's test on log transformed data (MC: n=12, MS: n=12, FC: n=12, FS: n=11; see figure for p-values). **b-c** This finding is supported by mass spectrometry data demonstrating a sex effect for total triglyceride-bound stearate (**b**), whereas sucrose intake increased newly elongated stearate in both sexes as determined by Scheirer-Ray-Hare test and post hoc Dunn test with Benjimini and Hochberg correction for multiple comparisons (MC: n=14, MS: n=14, FC: n=10, FS: n=13; see figure for p-values). **c** Both effects of sex and sucrose intake were observed for total triglyceride-bound oleate. Newly desaturated triglyceride-bound oleate was similarly increased in response to sucrose intake as determined in log transformed data by two-way ANOVA with post hoc Tukey's test (MC: n=14, MS: n=14, FC: n=10, FS: n=13; see figure for p-values). **d-e** Serum non-esterified fatty acids (NEFA) demonstrated a sex:sucrose interaction effect in response to lipolysis stimulation in vivo (**c**; basal/stimulated MC: n=11/11, MS: n=10/10, FC: n=7/6, FS: n=10/10; see figure for p-values), an effect no longer present in mice with genetically impaired lipolysis (**e**; control basal/stimulated MC: n=4/4, MS: n=6/6, FC: n=7/7, FS: n=8/8; knockout basal/stimulated MC: n=4/4, MS: n=6/6, FC: n=7/7, FS: n=8/8; *$p<0.05$). Data displayed are the group means ± standard error with biologically independent values overlayed. MC = Male Control, MS = Male Sucrose, FC = Female Control, FS = Female Sucrose. Source data are provided in the source data file.

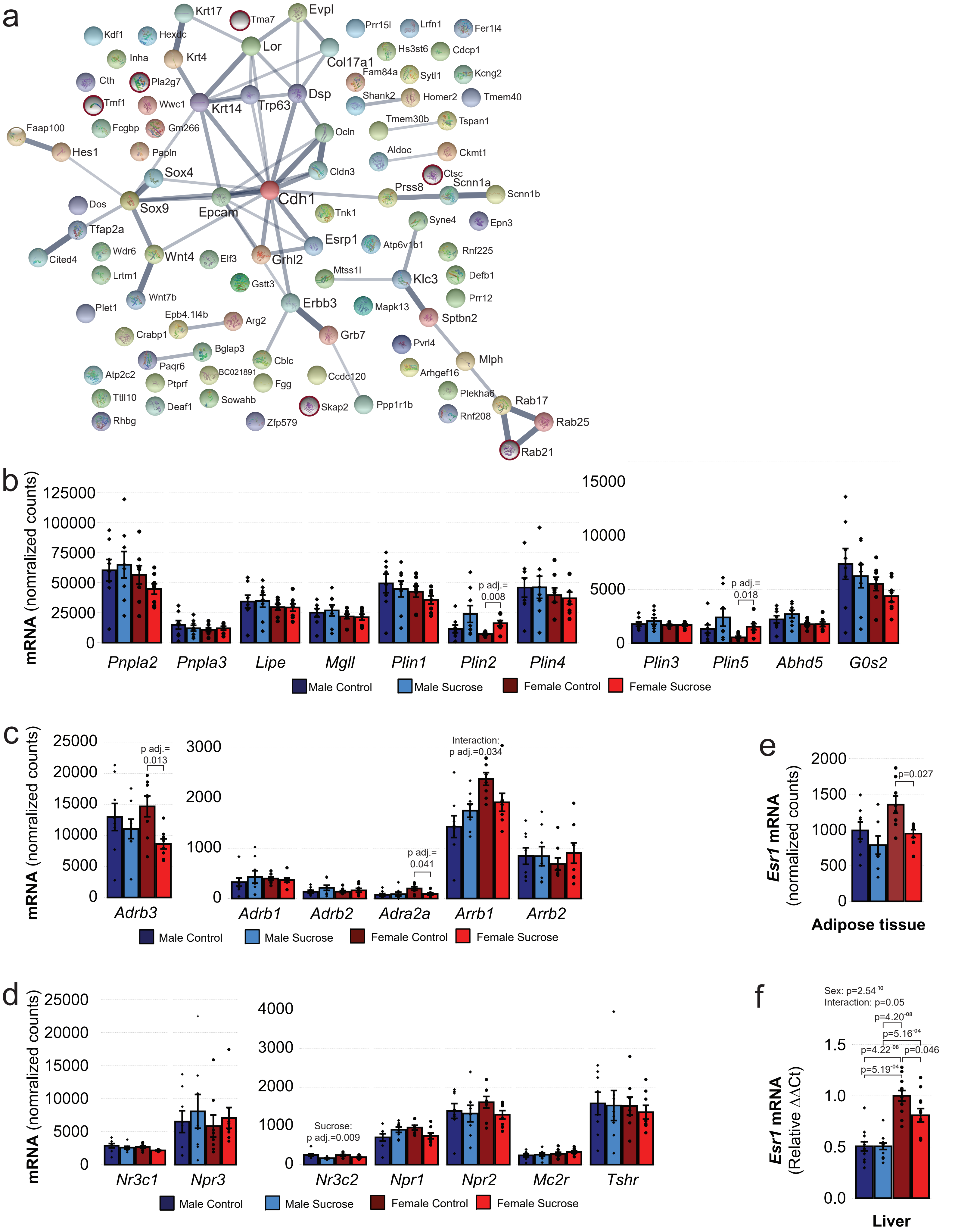

Supplementary Figure 4

Supplementary figure 4

String network analysis of differentially expressed genes common to both sexes, and lipolysis-related transcript expression

a String network analysis identified functional relationships between proteins encoded by gene transcripts that were commonly up- or down-regulated in both sexes. **b-d** The expression of transcripts encoding key lipolysis proteins or receptors known to be important for lipolysis signaling demonstrated a few female-specific effects, **c** with sucrose intake resulting in decreased expression of key adrenergic receptor signaling transcripts as determined by differential gene expression analysis (MC: n=8, MS: n=8, FC: n=8, FS: n=8; see figure for p-values. See also: Supplementary File 2). **b** Sucrose also affected the expression of transcripts encoding lipid droplet proteins **d** and the transcript encoding the mineralcorticoid receptor as determined by differential gene expression analysis (MC: n=8, MS: n=8, FC: n=8, FS: n=8; see figure for p-values. See also: Supplementary File 2), **e** whereas the transcript encoding the estrogen receptor α (*Esr1*) was reduced in both adipose tissue **f** (MC: n=8, MS: n=8, FC: n=8, FS: n=8; see figure for p-values. See also: Supplementary File 2) and liver as determined by two-way ANOVA with post hoc Tukey's test (MC: n=12, MS: n=12, FC: n=12, FS: n=11; see figure for p-values). Data displayed are the group means ± standard error with biologically independent values overlayed. MC = Male Control, MS = Male Sucrose, FC = Female Control, FS = Female Sucrose. Source data are provided in the source data file. Relative transcript expression, including Log2 fold change and adjusted p-values are available in Supplementary File 2. String network analysis data is available in Supplementary File 3.
