# Supplementary material for: Chronic intake of high dietary sucrose induces sexually dimorphic metabolic adaptations in mouse liver and adipose tissue
Source: Nat Commun. 2022 Oct 13;13:6062. doi: 10.1038/s41467-022-33840-6 (PMC9561177; doi:10.1038/s41467-022-33840-6)
Supplement: Supplementary file 2 — Description of Additional Supplementary Files [file 41467_2022_33840_MOESM2_ESM.pdf]

Description of Additional Supplementary Files

File Name: Supplementary Data 1
Description: Primer Sequences

File Name: Supplementary Data 2
Description: Differential Gene Expression (adipose tissue)

File Name: Supplementary Data 3
Description: String Network Analysis (adipose tissue)

File Name: Supplementary Data 4
Description: Gene Set Enrichment Analysis (adipose tissue)

File Name: Supplementary Data 5
Description: DESeq results (microbiome)
