# Supplementary material for: Chronic intake of high dietary sucrose induces sexually dimorphic metabolic adaptations in mouse liver and adipose tissue
Source: Nat Commun. 2022 Oct 13;13:6062. doi: 10.1038/s41467-022-33840-6 (PMC9561177; doi:10.1038/s41467-022-33840-6)
Supplement: Supplementary file 8 — Reporting Summary [file 41467_2022_33840_MOESM8_ESM.pdf]

nature portfolio

Corresponding author(s): Erin Stephenson

Last updated by author(s): Sep 25, 2022

Reporting Summary

Nature Portfolio wishes to improve the reproducibility of the work that we publish. This form provides structure for consistency and transparency in reporting. For further information on Nature Portfolio policies, see our Editorial Policies and the Editorial Policy Checklist.

Statistics

For all statistical analyses, confirm that the following items are present in the figure legend, table legend, main text, or Methods section.

n/a	Confirmed	
☐	☒	The exact sample size (*n*) for each experimental group/condition, given as a discrete number and unit of measurement
☐	☒	A statement on whether measurements were taken from distinct samples or whether the same sample was measured repeatedly
☐	☒	The statistical test(s) used AND whether they are one- or two-sided<br>*Only common tests should be described solely by name; describe more complex techniques in the Methods section.*
☐	☒	A description of all covariates tested
☐	☒	A description of any assumptions or corrections, such as tests of normality and adjustment for multiple comparisons
☐	☒	A full description of the statistical parameters including central tendency (e.g. means) or other basic estimates (e.g. regression coefficient) AND variation (e.g. standard deviation) or associated estimates of uncertainty (e.g. confidence intervals)
☐	☒	For null hypothesis testing, the test statistic (e.g. *F*, *t*, *r*) with confidence intervals, effect sizes, degrees of freedom and *P* value noted<br>*Give P values as exact values whenever suitable.*
☒	☐	For Bayesian analysis, information on the choice of priors and Markov chain Monte Carlo settings
☒	☐	For hierarchical and complex designs, identification of the appropriate level for tests and full reporting of outcomes
☒	☐	Estimates of effect sizes (e.g. Cohen's *d*, Pearson's *r*), indicating how they were calculated

Our web collection on statistics for biologists contains articles on many of the points above.

Software and code

Policy information about availability of computer code

Data collection	Oxymax software (v5.24) was used to collect data from CLAMS experiments. Tophat (2.1.1), STAR (v2.5), and HTSeq (v0.6.1) were used to collect and process RNA sequencing data.
Data analysis	Data analyses were performed using R (v4.1.1) running R Studio (build 492). Packages used for data processing, visualization and analysis included Tidyverse (v1.3.1), lubridate (v1.8.0), car (v3.1.0), lme4 (v1.1.29), rcompanion (v2.4.15), fsa (v0.9.3), DESeq2 (v1.24.0), fgsea (v1.10.1).String network analysis was performed using string-db.org (v11.0). Microbiome data were processed in QIIME (v1.9.1) using Calypso (v8.84).

For manuscripts utilizing custom algorithms or software that are central to the research but not yet described in published literature, software must be made available to editors and reviewers. We strongly encourage code deposition in a community repository (e.g. GitHub). See the Nature Portfolio guidelines for submitting code & software for further information.

Data

Policy information about availability of data

All manuscripts must include a data availability statement. This statement should provide the following information, where applicable:

- Accession codes, unique identifiers, or web links for publicly available datasets
- A description of any restrictions on data availability
- For clinical datasets or third party data, please ensure that the statement adheres to our policy

Gene set enrichment analyses performed for this work relied on the publicly available Molecular Signatures Database reference gene set (MSigDB.v7.0), available from https://www.gsea-msigdb.org/. Microbiome data generated for this project are available from the NCBI BioProject database, accession number PRJNA883667, https://www.ncbi.nlm.nih.gov/bioproject/883667. RNAseq data generated for this project are available from the NCBI Gene Expression Omnibus, accession number GSE151358, https://www.ncbi.nlm.nih.gov/geo/query/acc.cgi?acc=GSE151358. Other data generated for this work are provided in the Source Data file.

Human research participants

Policy information about studies involving human research participants and Sex and Gender in Research.

Reporting on sex and gender	*Use the terms sex (biological attribute) and gender (shaped by social and cultural circumstances) carefully in order to avoid confusing both terms. Indicate if findings apply to only one sex or gender; describe whether sex and gender were considered in study design whether sex and/or gender was determined based on self-reporting or assigned and methods used. Provide in the source data disaggregated sex and gender data where this information has been collected, and consent has been obtained for sharing of individual-level data; provide overall numbers in this Reporting Summary. Please state if this information has not been collected. Report sex- and gender-based analyses where performed, justify reasons for lack of sex- and gender-based analysis.*
Population characteristics	*Describe the covariate-relevant population characteristics of the human research participants (e.g. age, genotypic information, past and current diagnosis and treatment categories). If you filled out the behavioural & social sciences study design questions and have nothing to add here, write "See above."*
Recruitment	*Describe how participants were recruited. Outline any potential self-selection bias or other biases that may be present and how these are likely to impact results.*
Ethics oversight	*Identify the organization(s) that approved the study protocol.*

Note that full information on the approval of the study protocol must also be provided in the manuscript.

Field-specific reporting

Please select the one below that is the best fit for your research. If you are not sure, read the appropriate sections before making your selection.

☒ Life sciences ☐ Behavioural & social sciences ☐ Ecological, evolutionary & environmental sciences

For a reference copy of the document with all sections, see nature.com/documents/nr-reporting-summary-flat.pdf

Life sciences study design

All studies must disclose on these points even when the disclosure is negative.

Sample size	Pilot data obtained for liver triglyceride concentrations in male C57BL6/J mice after 12 weeks sucrose (10%w/v) consumption were used to determine power using the r package pwr. The study was powered to detect a >=60% increase in hepatic triglyceride content (alpha=0.05, beta=0.25). Sample sizes were adjusted for potential animal loss by adding at least 2 additional mice to the number generated by the power analysis.
Data exclusions	No data was excluded if available
Replication	The findings presented in the study replicate findings for liver triglyceride concentrations and adipose tissue transcript expression by qPCR obtained in pilot experiments in different cohorts of mice and, in some cases, in mice with different genetic backgrounds. Replicate experiments to verify findings were completed for western blots (gels/blots were ran twice on different days for each antibody and the results compared to ensure reproducibility) and qPCR experiments (a second set of cDNA was prepared and qPCR experiments were repeated once to ensure reproducibility of data). Each mouse (or its tissues) was considered a single replicate in all experiments and a minimum of 3 independent animals in both sexes/treatment groups were used for all assays to ensure reproducibility of findings. Exact numbers of mice in each group/analysis are described in the figure legends.
Randomization	Mice were randomly allocated to their treatment groups upon weaning

Blinding

Mice were each assigned a unique numeric identifier at 14 days of age (ear tag) and this identifier was used throughout the data collection and analysis process for all samples associated with that mouse. It was not possible to blind investigators during the in vivo data collection stages because mice receiving sucrose consumed more fluids than control mice which made it obvious which groups were consuming sucrose and which were consuming water. Genotype blinding was only possible for the control groups as the AdipoqCre-Pnpla2 mice were obese compared with the control groups and could be visually identified by investigators. For the majority of experiments performed on tissues, investigators only had access to the unique numeric identifier for each of the samples as data were generated. Exceptions were the RNAseq data and Mass Spectrometry data for which analyses were conducted by a commercial entity and core facility, respectively, where the group allocation information was a requirement upon submission of samples for processing and analysis.

Reporting for specific materials, systems and methods

We require information from authors about some types of materials, experimental systems and methods used in many studies. Here, indicate whether each material, system or method listed is relevant to your study. If you are not sure if a list item applies to your research, read the appropriate section before selecting a response.

Materials & experimental systems

n/a	Involved in the study	
☐	☒	Antibodies
☒	☐	Eukaryotic cell lines
☒	☐	Palaeontology and archaeology
☐	☒	Animals and other organisms
☒	☐	Clinical data
☒	☐	Dual use research of concern

Methods

n/a	Involved in the study	
☒	☐	ChIP-seq
☒	☐	Flow cytometry
☒	☐	MRI-based neuroimaging

Antibodies

Antibodies used

SREBP-1c clone IgG-2A4 (557036, BD Pharmingen, 1:1000), FAS clone C20G5 (3180 Cell Signaling Technology, 1:1000), ACC1/2 clone C83B10 (3676, Cell Signaling Technology, 1:1000), polyclonal phospho-ACC1/2 Ser79 (3661, Cell Signaling Technology, 1:1000), polyclonal ChREBP (NB400-135, Novus Biologicals, 1:1000), polyclonal PKLR (AV41699, Sigma-Aldrich, 1:200), beta-Tubulin clone BT7R (MA5-16308, Invitrogen, 1:2000), Goat anti-Rabbit IgG Alexa Fluor 680 secondary antibody (A21109, 1:15,000, Invitrogen) and Donkey anti-Mouse IgG Alexa Fluor 790 secondary antibody (A11371, 1:15,000, Invitrogen).

Validation

All antibodies were commercially purchased from established vendors, validated by the respective vendors or other researchers for use in western blotting, and cited in the literature and on citeab.com as having been used successfully by other investigators in the same species and application. We base specificity for the antibodies used on these reports as follows -

FAS: #3180 is a mAb with 384 citations. The technical data sheet from the manufacturer states that Fatty Acid Synthase (clone C20G5) detects endogenous levels of total fatty acid synthase protein and is validated for cross-reactivity in human, mouse, and rat tissues, and for use in western blotting. The manufacturer also states that species reactivity is determined by testing in at least one approved application (including western blot). https://www.cellsignal.com/datasheet.jsp?productId=3180&images=1

pACC: #3661 is a polyclonal Ab with 933 citations. The technical data sheet from the manufacturer states that Phospho-Acetyl-CoA Carboxylase (Ser79) Antibody detects endogenous levels of all isoforms of acetyl-CoA carboxylase protein and has been validated for cross-reactivity in human, mouse, rat, and hamster tissues, and for use in western blotting. The manufacturer also states that species reactivity is determined by testing in at least one approved application (including western blot). https://www.cellsignal.com/datasheet.jsp?productId=3676&images=1

ACC: #3676 is a mAb with 532 citations. The technical data sheet from the manufacturer states that Acetyl-CoA Carboxylase (clone C83B10) detects endogenous levels of ACC only when phosphorylated at serine 79. The antibody recognizes both ACCalpha and ACCbeta and has been validated for cross-reactivity in human, mouse, rat, and monkey tissues, and for use in western blotting. The manufacturer also states that species reactivity is determined by testing in at least one approved application (including western blot). https://www.cellsignal.com/datasheet.jsp?productId=3661&images=1

ChREBP: #NB400-135 is a polyclonal antibody with 125 citations. The technical data sheet from the manufacturer states that this polyclonal CHREBP Antibody is made from a C-terminal synthetic peptide made to the human CHREBP protein sequence (between residues 800-852). [UniProt# Q9NP71, Isoform 1/Alpha]. The manufacturers report that this antibody cross-reacts with human, mouse, and rat tissues and has been validated for use in western blotting. The manufacturer reports that this antibody has been cited for use with mouse tissues in 59 published manuscripts and for western blotting in 66 manuscripts https://www.novusbio.com/PDFs/NB400-135.pdf Since much of the published literature reporting use of this antibody for western blotting of mouse tissues fail to report the molecular weight, and our finding that the specific bands we observed appeared heavier than the molecular weight reported by the manufacturer, we validated the ChREBP antibody ourselves using a blocking peptide that the manufacturer claims to be specific to this antibody https://www.novusbio.com/products/chrebp-peptide_nb400-135pep.The blocking peptide completely blocked the bands we saw, indicating that the antibody was specific to ChREBP. The outcome of this validation is reported on the manufacturers website https://www.novusbio.com/products/chrebp-antibody_nb400-135#ReviewsSection:~:text=07/01/2021-,View,-Enlarge

PKLR: #AV41699 is a polyclonal Ab with 5 citations. The technical data sheet from the manufacturer states that this Anti-PKLR antibody produced in rabbit IgG fraction of antiserum is validated for use in western blotting and is cross-reactive to bovine, human,

rat, dog, mouse, rabbit, and pig tissues. The manufacturer reports that the immunogen was a synthetic peptide directed towards the N terminal region of human PKLR. This antibody is also mentioned as having been used on mouse tissues in the reporting summary for Zhou et al 2019 Nature 565 https://static-content.springer.com/esm/art%3A10.1038%2Fs41586-018-0749-z/MediaObjects/41586_2018_749_MOESM2_ESM.pdf

SREBP1: #557036 is a mAB with 31 citations. The technical data sheet for the SREBP1c antibody (clone clone IgG-2A4) states that this antibody is routinely tested for western blot. The manufacturer performs quality control testing using human tissues and the data sheet reports reactivity with hamster tissue https://www.bdbiosciences.com/content/bdb/paths/generate-tds-document.us.557036.pdf The antibody has been reported in the literature to cross react with mouse tissues, specifically mouse liver-
https://pubmed.ncbi.nlm.nih.gov/34248683/
https://pubmed.ncbi.nlm.nih.gov/32443556/
https://pubmed.ncbi.nlm.nih.gov/30821074/
https://pubmed.ncbi.nlm.nih.gov/29170467/

beta Actin: #MA5-16308 is a mAB with 95 citations. The technical data sheet for beta Actin (clone BT7R) states that it has been validated by the manufacturer for western blotting and species reactivity against Dog, Chicken, Human, Mouse, Non-human primate, Rabbit, and Rat. https://www.thermofisher.com/order/genome-database/dataSheetPdf?producttype=antibody&productsubtype=antibody_primary&productId=MA5-16308&version=251

Animals and other research organisms

Policy information about studies involving animals; ARRIVE guidelines recommended for reporting animal research, and Sex and Gender in Research

Laboratory animals	Mus musculus, cross of mice congenic to C57BL6/N and C57BL6/J backgrounds, both males and females studied, multiple ages studied (range 4-23 weeks of age), originating strains obtained from The Jackson Laboratories B6N.129S-Pnpla2tm1Eek/J, stock number 944 024278 and B6FVB-Tg(Adipoq-cre)1Evdr/J, stock number 02802. All mice studied were from the same generation. Mice were group housed in ventilated cages in a temperature and humidity controlled environment at ~22 deg C with a 12:12 h light/dark cycle. Mice had free access to food (Envigo #7912) and water (or water containing 10% w/v sucrose).
Wild animals	No wild animals were used in this study
Reporting on sex	Both male and female mice were studied and compared in each of the experiments reported in this manuscript
Field-collected samples	No field collected samples were used in this study
Ethics oversight	UTHSC Institutional Animal Care and Use Committee approval approved the research prior to the work being completed (protocol #3180)

Note that full information on the approval of the study protocol must also be provided in the manuscript.
